# *N*-glycosylation modulates enzymatic activity of *Trypanosoma congolense* trans-sialidase

**DOI:** 10.1016/j.jbc.2022.102403

**Published:** 2022-08-20

**Authors:** Jana Rosenau, Isabell Louise Grothaus, Yikun Yang, Nilima Dinesh Kumar, Lucio Colombi Ciacchi, Sørge Kelm, Mario Waespy

**Affiliations:** 1 Faculty for Biology and Chemistry, Centre for Biomolecular Interactions Bremen, University of Bremen, Bremen, Germany; 2 Hybrid Materials Interfaces Group, Faculty of Production Engineering, Bremen Center for Computational Materials Science, Center for Environmental Research and Sustainable Technology (UFT), and MAPEX Center for Materials and Processes, University of Bremen, Bremen, Germany

**Keywords:** proteoglycan, molecular dynamics, mass spectrometry, enzyme kinetics, CD, *N*-glycosylation, protein–glycan interactions, trans-sialidases, *trypanosoma*, 3′SL, 3′-sialyllactose, CHO, Chinese hamster ovary, ConA, concanavalin A, EndoH_f_, endoglycosidase H, GlcNAc, N-acetylglucosamine, GPI, glycosylphosphatidylinositol, HPAEC, high-performance anion exchange chromatography, H-TconTS1, hypoglycosylated TconTS1, MD, molecular dynamics, MS, mass spectrometry, PAD, pulsed amperometric detection, PDB, Protein Data Bank, Sia, sialic acid, TconTS1, *Trypanosoma congolense* trans-sialidase 1, TcruTS, *Trypanosoma cruzi* trans-sialidase, TranSA, *Trypanosoma rangeli* sialidase, TS, trans-sialidase

## Abstract

Trypanosomes cause the devastating disease trypanosomiasis, in which the action of trans-sialidase (TS) enzymes harbored on their surface is a key virulence factor. TS enzymes are *N*-glycosylated, but the biological functions of their glycans have remained elusive. In this study, we investigated the influence of *N*-glycans on the enzymatic activity and structural stability of TconTS1, a recombinant TS from the African parasite *Trypanosoma congolense*. We expressed the enzyme in Chinese hamster ovary Lec1 cells, which produce high-mannose type *N*-glycans similar to the TS *N*-glycosylation pattern *in vivo*. Our MALDI-TOF mass spectrometry data revealed that up to eight putative *N*-glycosylation sites were glycosylated. In addition, we determined that *N*-glycan removal *via* endoglycosidase H_f_ treatment of TconTS1 led to a decrease in substrate affinity relative to the untreated enzyme but had no impact on the conversion rate. Furthermore, we observed no changes in secondary structure elements of hypoglycosylated TconTS1 in CD experiments. Finally, our molecular dynamics simulations provided evidence for interactions between monosaccharide units of the highly flexible *N*-glycans and some conserved amino acids located at the catalytic site. These interactions led to conformational changes, possibly enhancing substrate accessibility and enzyme–substrate complex stability. The here-observed modulation of catalytic activity *via N*-glycans represents a so-far-unknown structure–function relationship potentially inherent in several members of the TS enzyme family.

*N*-linked glycosylation is the attachment of an oligosaccharide to an asparagine residue (*N*-glycan) at the general sequon type Asn-Xaa-Ser/Thr and widespread in proteins of eukaryotes. *N*-glycans are involved in folding and stability of proteins and protect against proteolysis. In addition, *N*-glycans play an important role in regulating protein functions, represent target structures for lectins or antibodies, and can function as mediators of cell–matrix interactions and cell–cell recognition and communication (1, 2). A number of recent studies provide increasing evidence that *N*-glycans affect protein function by modulating substrate binding and turnover. For example, it has been demonstrated that *N*-glycans alter substrate affinity and turnover of several human proteases including metalloproteases (3) and serine proteases (4, 5, 6). In an early work, Wittwer *et al.* (4) have demonstrated that the presence and diversity of *N*-glycosylation modulates activity and function of the serine protease tissue-type plasminogen activator *in vitro*. They found that occupancy and nature of *N*-glycans attached to tissue-type plasminogen activator affect substrate binding and enzyme activity in a very specific manner.

In this study, we investigated the influence of *N*-glycans on the catalytic transfer activity, thermal stability, and structural conformation of *Trypanosoma congolense* trans-sialidase (TconTS) 1, by means of enzyme kinetics, mass spectrometry (MS) measurements, and molecular dynamics (MD) simulations. TSs are unusual enzymes expressed by different species of the parasitic genus *Trypanosoma*. This includes human pathogens causing Chagas disease in South America (*Trypanosoma cruzi*) and human African trypanosomiasis, also known as sleeping sickness, in Africa (*Trypanosoma brucei*), as well as animal pathogens being responsible for the animal African trypanosomiasis (mainly *T. brucei brucei*, *T. congolense*, and *Trypanosoma vivax*) (7, 8, 9, 10). The glycosylphosphatidylinositol (GPI)-anchored enzymes preferentially transfer α2,3-linked sialic acids (Sia) from host–cell glycoconjugates to terminal β-galactose residues of glycoproteins present on the parasite’s surface, thus creating a new α2,3-glycosidic linkage (7, 8, 11). This surface sialylation has different beneficial functions for the parasite, which is unable to synthesize Sia *de novo*. In particular, it promotes survival in the insect vector and enables to escape the host’s immune system (12, 13, 14, 15, 16). Thus, TSs are important virulence factors and represent promising drug targets and vaccine candidates to combat the fatal diseases caused by trypanosomes.

The enzymatic mechanisms and biological functions of TS have been under study for many years, although little attention has been drawn to the influence of post-translational modifications such as glycosylation (17, 18, 19, 20, 21, 22).

Interestingly, the presence of high-mannose type *N*-glycans has been inferred indirectly by concanavalin A (ConA) purification for many TSs (8, 23, 24). On the other hand, hybrid *N*-glycans or complex *N*-glycans have not been reported for TS. It has been postulated that *N*-glycans are involved in TS oligomerization (25). However, their influence on enzyme activity is still debated (17, 18, 21, 22). Previous experiments were performed with recombinant TS expressed by *Pichia pastoris*, which produces hypomannosylated *N*-glycans (21, 22). However, trypanosomal surface proteins were reported to harbor shorter high-mannose type *N*-glycans (26, 27, 28, 29, 30, 31, 32, 33). No differences in Sia transfer activity were observed between glycosylated and deglycosylated recombinant TS expressed by *P. pastoris* (21, 22) even though kinetic parameters were not determined.

Recombinant TconTS1 from the animal pathogen *T. congolense* expressed in Chinese hamster ovary (CHO) Lec1 cells was used in this study (34). Natively, TconTS1 is expressed by procyclic insect-infective trypanosomes as well as bloodstream-form trypanosomes in mammalian hosts and is involved in desialylation of erythrocytes, which contributes to anemia (35, 36). Like all TSs, TconTS1 consists of an N-terminal catalytic domain responsible for the transfer of Sia and of a C-terminal lectin-like domain whose biological function remains unclear. Recently, we demonstrated that the TconTS lectin-like domain can modulate enzymatic activity (37). The catalytic and lectin-like domain are connected *via* an α-helix (Fig. 1) (17, 18, 34). The N-terminus includes a signal sequence, whereas the C-terminus comprises a potential GPI anchor attachment site (34). So far, 17 TconTS-like genes have been described for *T. congolense*, from which 11 can be grouped into the TconTS1 family because of their high amino acid sequence identity (>96%). TconTS1b was chosen in this study, because it has been isolated from procyclic trypomastigotes (38) and possesses one of the highest enzyme activity among the TconTS family (34, 39) and is simply called TconTS1 onward in this article.

**Figure 1:**
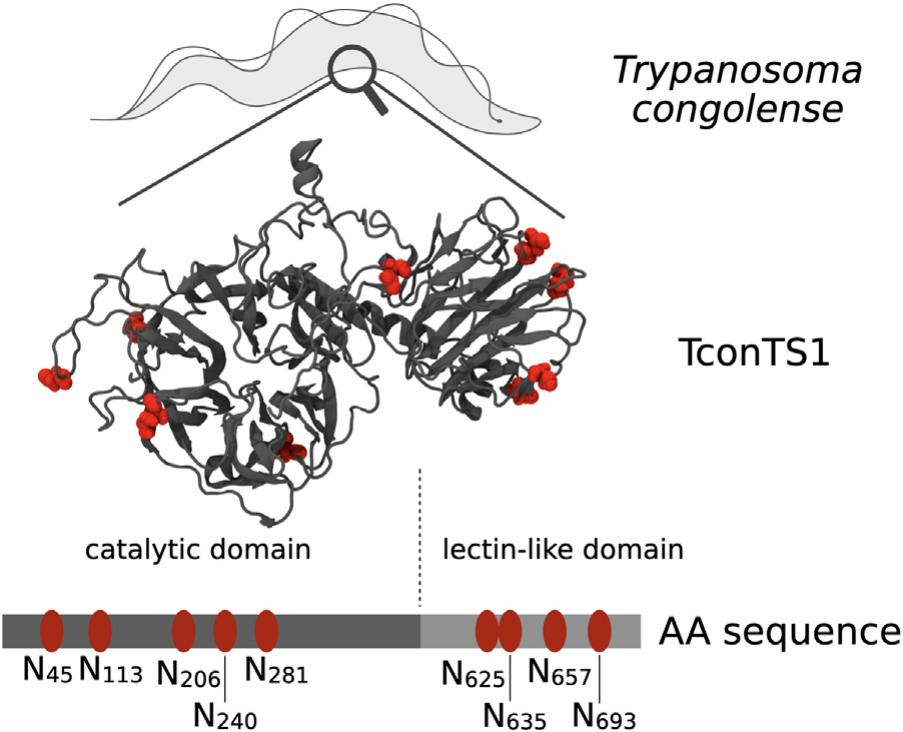
**Molecular model of TS originating from *Trypanosoma congolense* 1 (TconTS1) with *N*-glycosylation sites.** Asparagine residues in the motif N-X-S/T as putative *N*-glycosylation sites are highlighted in *red*. The positions of each asparagine are labeled in the amino acid sequence and sorted into the catalytic domain (CD) or the lectin-like domain (LD). TS, trans-sialidase.

An important structural feature of TconTS1 is the predicted nine *N*-glycosylation recognition sequences (Fig. 1), distributed across both the catalytic and lectin-like domains (34). It is noteworthy that TS sequences from African trypanosomal species contain a higher number of putative *N*-glycosylation sites (6, 7, 8, 9) compared with those of species from South American trypanosomes (2, 3, 4, 5) including the structural closely related *Trypanosoma rangeli* sialidase (TranSA) (10, 21, 34, 39, 40, 41, 42). Crystallization of TranSA revealed that all five potential *N*-glycosylation sites were occupied with *N*-glycans, although only the innermost monosaccharide could be detected, giving no hint about the type of *N*-glycosylation (17). The only crystallized TS from *T. cruzi*, however, was a recombinant protein containing several mutations in the amino acid sequence (18). Thus, the *N*-glycosylation pattern of this TS remains unresolved. In contrast to *T. brucei* (43), there are no database entries on potential oligosaccharyltransferase isoforms in *T. congolense*. Therefore, no further assessment of potential glycosylation pattern and glycan donor–acceptor and peptide–acceptor specificity could be made so far.

To investigate *N*-glycosylation-dependent structure–function effects, we expressed recombinant TconTS1 in leucophytohemagglutinin-resistant Lec1 CHO cells (44, 45). This *N*-glycosylation mutant cell line is unable to synthesize complex and hybrid *N*-glycans and consequently accumulates high-mannose type *N*-glycans of the composition Man_5–9_GlcNAc_2_. This mimics the situation reported for African trypanosomes, which preferentially synthesize oligosaccharides bearing Man_5–9_GlcNAc_2_ *N*-glycans (26, 27, 28, 29, 30, 31, 44, 45). For these reasons, we have selected the CHO Lec1 cell line as an *N*-glycosylation model system in this study. The presence and composition of *N*-glycans at the putative *N*-glycosylation sites in TconTS1 was analyzed qualitatively by MALDI-TOF MS, in order to identify modified sites and oligosaccharide distribution. Enzyme activities of glycosylated and endoglycosidase H_f_ (EndoH_f_)-treated TconTS1 were analyzed *via* quantification of reaction products using high-performance anion exchange chromatography (HPAEC) with pulsed amperometric detection (PAD). CD spectroscopy was used to compare the secondary structures and stabilities of glycosylated *versus* EndoH_f_-treated TconTS1. Finally, MD simulations were performed to characterize potential protein–glycan and glycan–glycan interactions altering the dynamics of amino acids in close proximity to the catalytic domain.

## Results

### Mapping the *N*-glycosylation pattern of TconTS1

**Figure 2:**
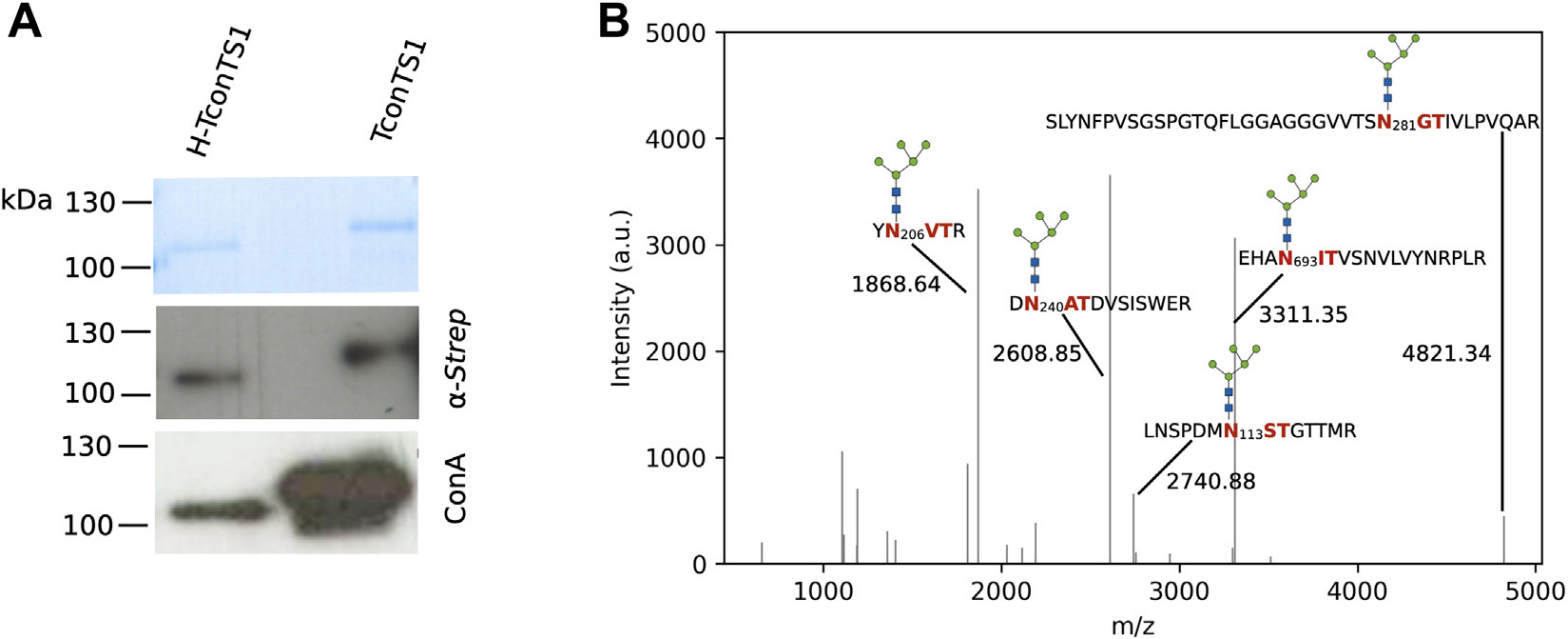
**Mapping the *N*-glycosylation profile of untreated TconTS1 and hypoglycosylated TconTS1 (H-TconTS1).***A*, TconTS1 and H-TconTS1 (treated with EndoH_f_ for 4 h) were analyzed by SDS-PAGE with subsequent Coomassie staining (*upper panel* with 600 ng of protein), Western blot analysis using an anti-*Strep*-tag antibody (*middle panel* with 400 ng of protein), and lectin blotting using ConA (*lower panel* with 100 ng of protein). Exposure time of Western blot is 5 and 60 s for the ConA blot. Results indicate the presence of high-mannose type *N*-glycans. *B*, MALDI-TOF MS analyses were performed to identify *N*-glycosylation sites of TconTS1. Glycopeptides from protease-digested TconTS1 were ConA purified to concentrate glycopeptides and reduce the spectrum complexity. Peak lists were extracted from MALDI-TOF mass spectra, plotted with python, and annotated with corresponding masses and glycopeptide fragments, respectively. Monosaccharide symbols follow the symbol nomenclature for glycans (92). MS, mass spectrometry; TconTS1, *Trypanosoma congolense* trans-sialidase 1.

To characterize the *N*-glycosylation pattern of TconTS1 in detail, we expressed TconTS1 in CHO Lec1 cells (Figs. S1*A* and S2) (34). High-mannose type *N*-glycans of the recombinant protein were detected by ConA lectin blots (Figs. 2*A* and S3, *A* and *B*). For the selective removal of *N*-glycans, TconTS1 was treated with EndoH_f_. A clear band shift of approximately 10 kDa for TconTS1 after 4 h of incubation with EndoH_f_ was observed, indicating a size reduction as a consequence of the removal of *N*-glycans from TconTS1 (Figs. 2*A* and S4, *A* and *B*). In addition, less binding of ConA to EndoH_f_-treated TconTS1 was observed by lectin blots, indicating a loss of *N*-glycan structures (Fig. 2*A*, *lower panel*). However, deglycosylation was not complete after 4 h of incubation, and even further addition of EndoH_f_ or increased incubation times to up to 48 h did not result in complete deglycosylation. EndoH_f_-treated TconTS1 incubated overnight (16 h) (Fig. S4, *C* and *D*) was used for *N*-glycosylation pattern analysis in this study. This modified TconTS1 is termed hypoglycosylated TconTS1 (H-TconTS1) onward. Raw data and further details of Western and lectin blot analysis are given in Section S2 of the supporting information.

MALDI-TOF MS experiments were performed to identify *N*-glycan structures and the distribution pattern. In order to analyze TconTS1 and H-TconTS1 by MALDI-TOF MS, both enzymes were proteolytically digested to shorter peptide and glycopeptide fragments. Whenever an asparagine residue in the N-X-S/T motif of a certain glycopeptide is glycosylated, the *m/z* ratio increases by exactly the mass of the conjugated *N*-glycan relative to the nonglycosylated peptide.

We employed different analyses with varying sample preparations to crossvalidate identified *N*-glycans of TconTS1. First, we used two different proteases, trypsin and chymotrypsin, to generate glycopeptides. An advantage of this strategy is that treatment with either trypsin or chymotrypsin results in different glycopeptide profiles because of their different specific protease recognition sites (for trypsin digestion, see Fig. S5*B*). As a result, the same potential *N*-glycosylation site can be found within different glycopeptides after either trypsin or chymotrypsin digestion. Second, glycopeptides were purified using ConA sepharose to specifically enrich and concentrate glycopeptides comprising high-mannose type *N*-glycans and as a consequence to lower the complexity of spectra (Figs. 2*B* and S5*A*). Third, H-TconTS1 was analyzed using the same strategy described previously. EndoH_f_ cleaves within the chitobiose core of high-mannose type *N*-glycans, leaving a residual N-acetylglucosamine (GlcNAc) at the glycopeptide. Thus, peptides generated from H-TconTS1 that comprise a putative *N*-glycosylation site and exhibit a mass difference of *m/z* 203.08 (one HexNAc, corresponding to one GlcNAc) relative to the nonglycosylated peptide indicate the presence of a high-mannose type *N*-glycan at that site (Fig. S5*C*). A summarizing evaluation of these different approaches is given in Table 1.

**Table 1:** MALDI-TOF MS analyses of the *N*-glycan profile of TconTS1

Treatment	*N*-glycan	N_45_	N_113_	N_206_	N_240_	N_281_	N_625_	N_635_	N_657_	N_693_
Trypsin	Nonglycosylated				G					
	Man_5_GlcNAc_2_		G	G	G	G				G
	Man_6_GlcNAc_2_									
	Man_7_GlcNAc_2_									
	Man_8_GlcNAc_2_									
	Man_5_GlcNAc_2_Fuc1			G						
	HexNAc		H	H	H	H^a^				H
Trypsin + ConA	Man_5_GlcNAc_2_		G	G/H	G	G				G
	Man_6_GlcNAc_2_			G/H						H^a^
	Man_7_GlcNAc_2_			G						
	Man_8_GlcNAc_2_			H						
	Man_5_GlcNAc_2_Fuc1									
Chymotrypsin	Nonglycosylated		G	G			G/H			H
	Man_5_GlcNAc_2_	G	H^a^	G						
	Man_6_GlcNAc_2_		G^a^							
	Man_7_GlcNAc_2_									
	Man_8_GlcNAc_2_									
	Man_5_GlcNAc_2_Fuc1									
	HexNAc	H		H					H	H
Chymotrypsin + ConA	Man_5_GlcNAc_2_	G		G/H					G	
	Man_6_GlcNAc_2_			G/H						
	Man_7_GlcNAc_2_									
	Man_8_GlcNAc_2_			G/H						
	Man_5_GlcNAc_2_Fuc1									

For analysis, the untreated TconTS1 (glycosylated, G) and the EndoH_f_-treated H-TconTS1 (H) were digested either with trypsin or chymotrypsin. Spectra were analyzed for masses corresponding to glycopeptides with high-mannose type *N*-glycans and for nonglycosylated peptides with potential *N*-glycosylation sites. Spectra of H-TconTS1 were in addition analyzed for glycopeptides with HexNAc residues since a residual GlcNAc remains attached to the protein *N*-glycosylation sites after EndoH_f_ treatment. In another approach, glycopeptides from both proteins were purified with ConA after protease digestion, and spectra were analyzed for masses of peptides with high-mannose type *N*-glycans. Fuc, fucose; GlcNAc, N-acetylglucosamine; Man, mannose.

a Glycopeptide mass was only detected once during multiple analyses.

Glycopeptides comprising high-mannose type *N*-glycans were detected especially in the catalytic domain. *N*-glycans attached to N113 and N206 were detected in both trypsin-treated and chymotrypsin-treated TconTS1 (Table 1). The mass difference corresponding to an *N*-glycan of the composition Man_5_GlcNAc_2_ was predominantly detected at N113, whereas N206 also exhibited the composition Man_6–8_GlcNAc_2_ (Tables 1 and S2). In addition, when analyzing H-TconTS1, HexNAc residues were detected at positions N113 and N206. But for both sites also, corresponding nonglycosylated peptides were detected. Interestingly, oligomannosidic *N*-glycosylation at N206 was still detectable after 16 h of EndoH_f_ treatment. This finding is supported by our ConA lectin blot results, demonstrating the binding of ConA to H-TconTS1 at long exposure times (Figs. 2 and S4).

Glycopeptides containing high-mannose type *N*-glycans conjugated to other *N*-glycosylation sites were detected either in the trypsin-treated or chymotrypsin-treated samples. For example, the mass difference corresponding to the composition Man_5_GlcNAc_2_ was identified at N45 and N657 (chymotrypsin) and at N240, N281, and N693 (trypsin) (Table 1). In addition, the same glycopeptides were identified in the corresponding ConA-purified samples (Table 1). When analyzing H-TconTS1, glycopeptides with mass differences corresponding to a residual HexNAc residue were detected in the glycopeptides obtained after digestion with the corresponding proteases (Table 1). N240 and N693 (only in H-TconTS1) were also detected in their nonglycosylated forms, indicating a heterogeneity at these sites. Peptides containing N625 were only found as the nonglycosylated form. Additional details regarding the performed MALDI-TOF MS analysis can be found in Section S3 of the supporting information.

In summary, the compiled data have revealed *N*-glycosylation predominantly in the catalytic domain of recombinant TconTS1 allowing for a qualitative prediction of the predominant *N*-glycan composition for different *N*-glycosylation sites.

### *N*-glycosylation of TconTS1 influences the affinity to the Sia acceptor substrate in the transfer reaction

To test the impact of *N*-glycosylation on enzyme activity, we performed activity assays using H-TconTS1 and TconTS1. For these assays, TconTS1 was exposed to identical conditions applied during the EndoH_f_ treatment. Fetuin and lactose were used as Sia donor and acceptor substrates in enzyme reactions, as previously described (34, 39). The transfer reaction product 3′-sialyllactose (3′SL) was quantified by HPAEC–PAD. This HPLC-based method allows for the separation and detection of the educt lactose and the product 3′SL. Incubation times of 30 min and 600 μM fetuin-bound Sia were used in these experiments. These conditions were chosen in order to compare our results with those already available in the literature (34). The Sia concentration used corresponds approximately to one-third of the Sia concentration reported to be present on glycoproteins in blood serum (46, 47). In human blood serum, a high proportion of protein-bound Sia is α2,6-linked (48, 49). A lactose concentration series was applied in order to calculate the corresponding Michaelis–Menten kinetic parameters *K*_*M*_ and *V*_max_.

**Figure 3:**
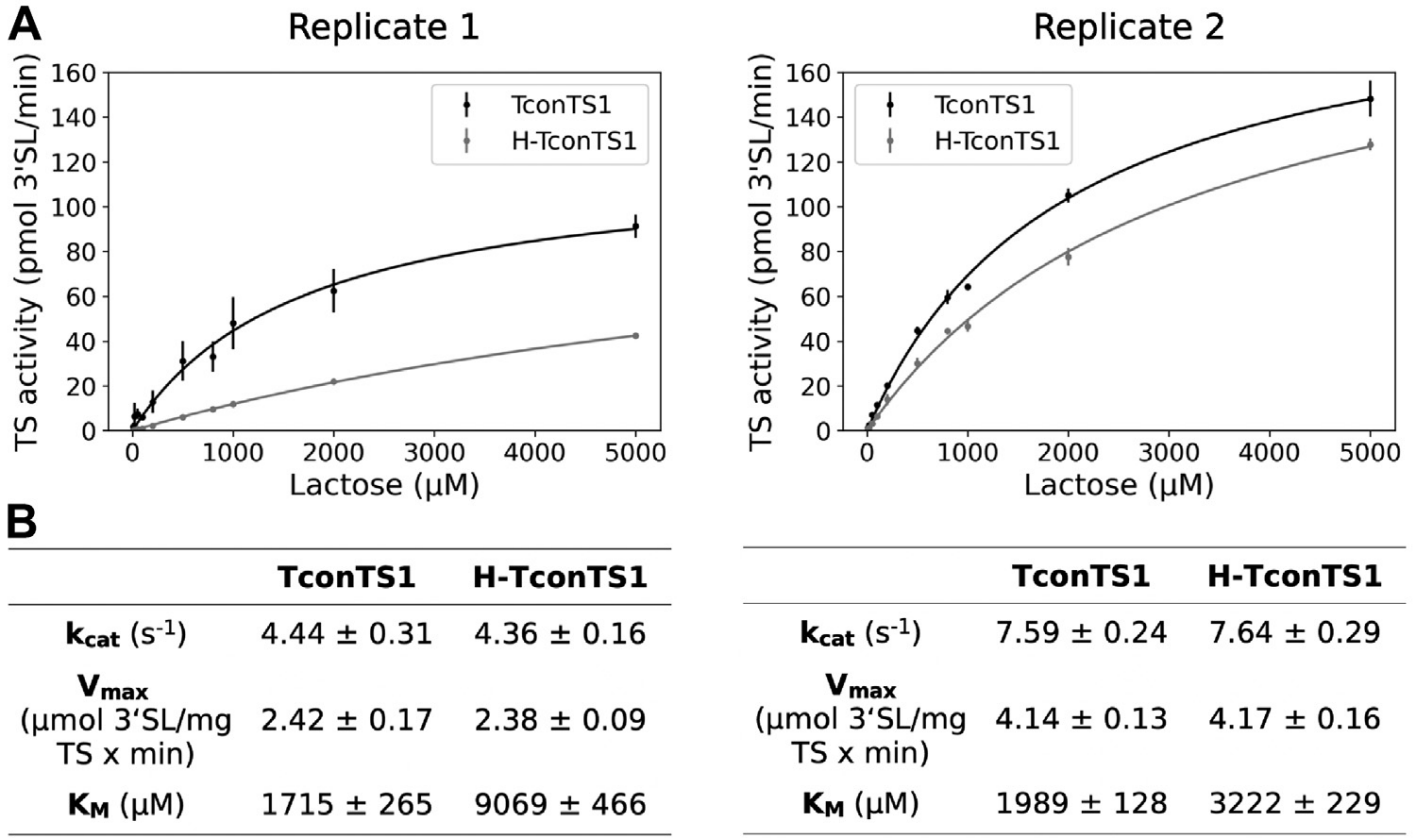
**EndoH**_**f**_**-treated H-TconTS1 shows lower *K***_***M***_**compared with TconTS1.***A* and *B*, TS activities for TconTS1 and H-TconTS1 were determined using fetuin as Sia donor and a lactose concentration series as Sia acceptor. Production of 3′-sialyllactose was monitored and Michaelis–Menten kinetic parameters, apparent *K*_*M*_ and *V*_max_, as well as *k*_cat_ for lactose were evaluated using SigmaPlot11. Data points are means ± standard deviation of three technical replicates for each biological replicate. H-TconTS1, hypoglycosylated TconTS1; TconTS1, *Trypanosoma congolense* trans-sialidase 1; TS, trans-sialidase.

Two different TconTS1 preparations (biological replicates) and the corresponding hypoglycosylated proteins were investigated. Both datasets revealed that H-TconTS1 produced lower amounts of 3′SL relative to TconTS1 in these experiments (Fig. 3*A*). Interestingly, the calculated *V*_max_ values for the Sia acceptor substrate lactose of about 2.4 μmol 3′SL/(min × mg enzyme) for replicate 1 and 4.1 μmol 3′SL/(min × mg enzyme) for replicate 2 were found to be identical within the error range in both datasets (Fig. 3*B*). These results indicate that the 3′SL production rate of TconTS1 at saturated Sia acceptor concentrations is not influenced by its *N*-glycosylation state. Differences in *V*_max_ values between the datasets might be explained by varying amounts of active enzyme after purification. However, to study the influence of *N*-glycosylation on enzyme activity, the kinetic parameters were calculated separately for each replicate.

Interestingly, the *K*_*M*_ values for lactose differed between TconTS1 (1.7 and 2.0 mM) and H-TconTS1 (9.0 and 3.2 mM), indicating a 1.6-fold to 5-fold lower Sia acceptor substrate affinity for H-TconTS1 relative to TconTS1 (Fig. 3). The *K*_*M*_ values determined in this study are similar to the one published by Koliwer-Brandl *et al.* (34) (1.7 mM). Variations in the *K*_*M*_ of H-TconTS1 between replicates might be a result of differences in the number of *N*-glycans remaining on these hypoglycosylated TconTS1 preparations, as slight variations in the purification process are likely.

In summary, the same trend was observed for both datasets: an increase in the *K*_*M*_ value for lactose after EndoH_f_ treatment, whereas the *V*_max_ value remained constant.

### EndoH_f_ treatment of TconTS1 does not alter the overall secondary structure

Once we were able to determine the *N*-glycan profile of TconTS1 and its influence on the enzyme activity, we investigated whether the removal of *N*-glycans affects the enzyme stability. Since the presence of *N*-glycans can impact the protein’s function *via* altering the protein structure (50), we performed CD experiments to investigate the influence of *N*-glycans on the secondary structure stability of TconTS1.

**Figure 4:**
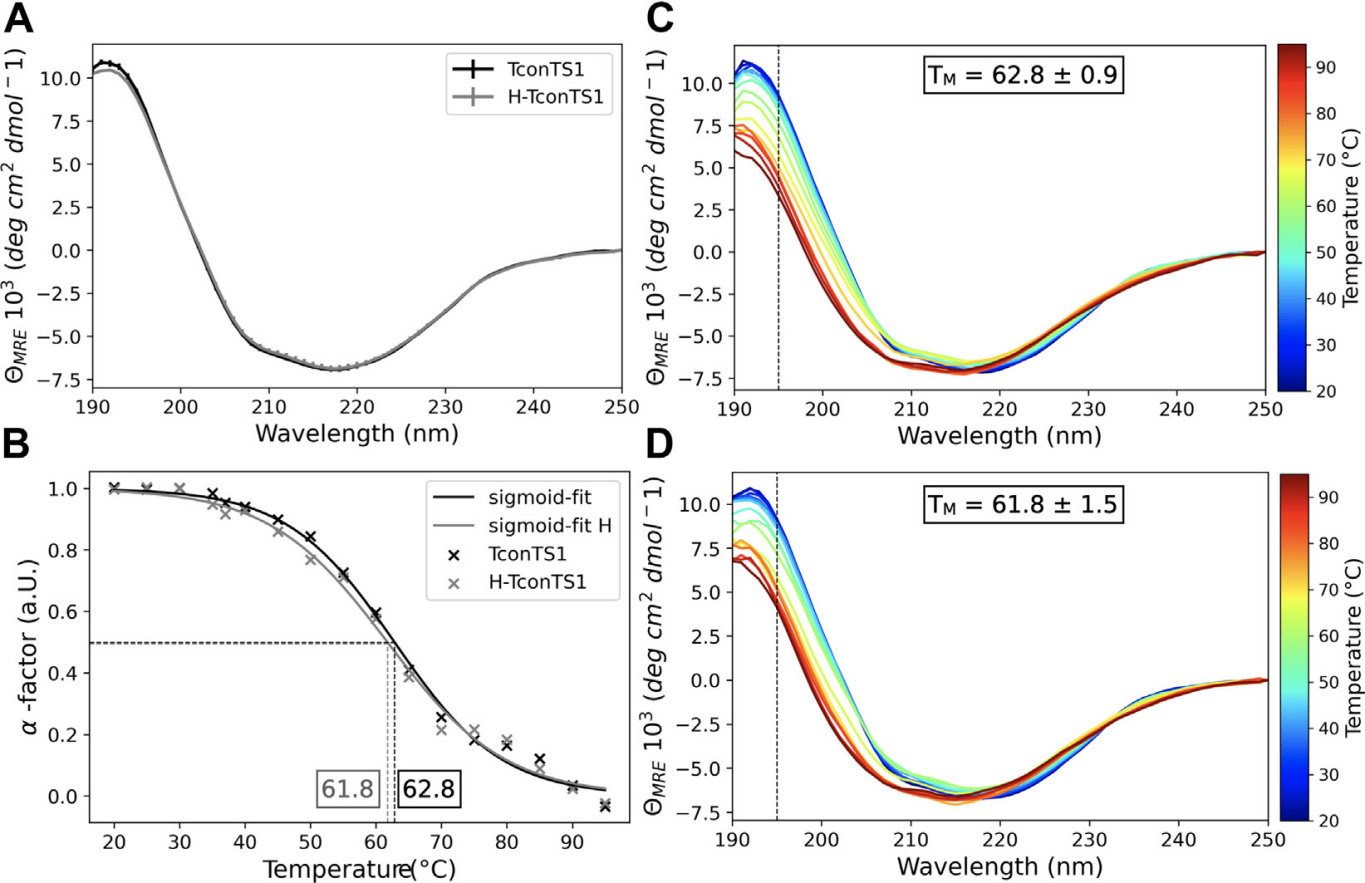
**Influence of *N*-glycans on TconTS1 secondary structure and stability.***A*, CD spectra of untreated TconTS1 and EndoH_f_-treated H-TconTS1 of replicate 1 were measured at 35 °C in 10 mM phosphate buffer (pH 7.4) (means ± standard deviation of two biological replicates). *B*, the midpoint of unfolding (TM = α-factor = 0.5) from a folded state (α-factor ∼1.0 at 20 °C) to a partially unfolded intermediate (α-factor ∼0.0 at 95 °C) was determined for TconTS1 and H-TconTS1 by fitting a sigmoid function to the data. CD spectra of temperature-ramping experiments with TconTS1 (*C*) and H-TconTS1 (*D*) were recorded in 5 °C steps and a 5 min equilibration time at each step. The midpoint of unfolding (TM) is determined at 195 nm (*dashed line*) at the flex point of a sigmoidal function fitting the temperature curve. H-TconTS1, hypoglycosylated TconTS1; TconTS1, *Trypanosoma congolense* trans-sialidase 1.

CD spectra of TconTS1 and H-TconTS1 were analyzed under similar conditions used for the enzyme activity measurements (35 °C) (Fig. 4*A*). The recorded spectra did not show any significant difference over the recorded wavelength range, indicating that both enzymes share the same common secondary structure. In fact, calculated secondary structural elements were identical in both cases, with 37% of β-sheets, 13% of α-helices, 11% of turns, and 39% of unstructured (other) components (Table S3).

Spectra recorded during temperature-ramping experiments revealed a high heat stability for both protein preparations, as TconTS1 and H-TconTS1 still kept intact secondary structural elements up to 95 °C (Fig. 4, *C* and *D*). The variation of the spectra intensity in the range between 190 and 210 nm during heating indicates a partial unfolding, taking place between 60 and 70 °C, with a melting temperature (*T*_M_) of about 62 °C for both proteins (Fig. 4*B*). Thus, TconTS1 and H-TconTS1 exhibit the same secondary element distribution and the same heat stability (as quantified by *T*_M_ for partial unfolding).

In summary, the CD experiments did not provide evidence for an altered overall secondary structure of H-TconTS1 as an explanation for its lower substrate affinity observed. However, *N*-glycosylation-induced changes of the tertiary structure remain as possible explanation because they are too subtle to be detected by this method.

### Structural importance of TconTS1 *N*-glycans indicated by MD simulations

#### The *N*-glycan shield

MD simulations can facilitate the study of mechanisms at the atomistic level and were performed to obtain an in-depth picture of *N*-glycan–protein interactions in TconTS1. Because of the lack of experimentally derived structures for TconTS enzymes, homology models were generated on the basis of TranSA and TcruTS. Further details about the model construction are given in the “Experimental procedures” section (see also Section S5 of the supporting information). Although TconTS1 only shares an amino acid sequence identity of 48% with TcruTS, both enzymes reveal a high overall structural similarity (Fig. S6) and only differ in three amino acids reported to be important for TS activity (34). In order to generate an *N*-glycosylated structural model of TconTS1, Man_5_GlcNAc_2_ glycans were included at positions N45, N113, N206, N240, N281, and N693, as identified by our MALDI-TOF MS experiments. In this model, all potential *N*-glycosylation sites are glycosylated in the catalytic domain, whereas only one of four potential sites is glycosylated in the lectin-like domain (Fig. S7*A*). In the model used to simulate H-TconTS1, single GlcNAc residues were included at positions that were also occupied in the TconTS1 model, mimicking the residual monosaccharide after EndoH_f_ treatment (Fig. S7*B*).

**Figure 5:**
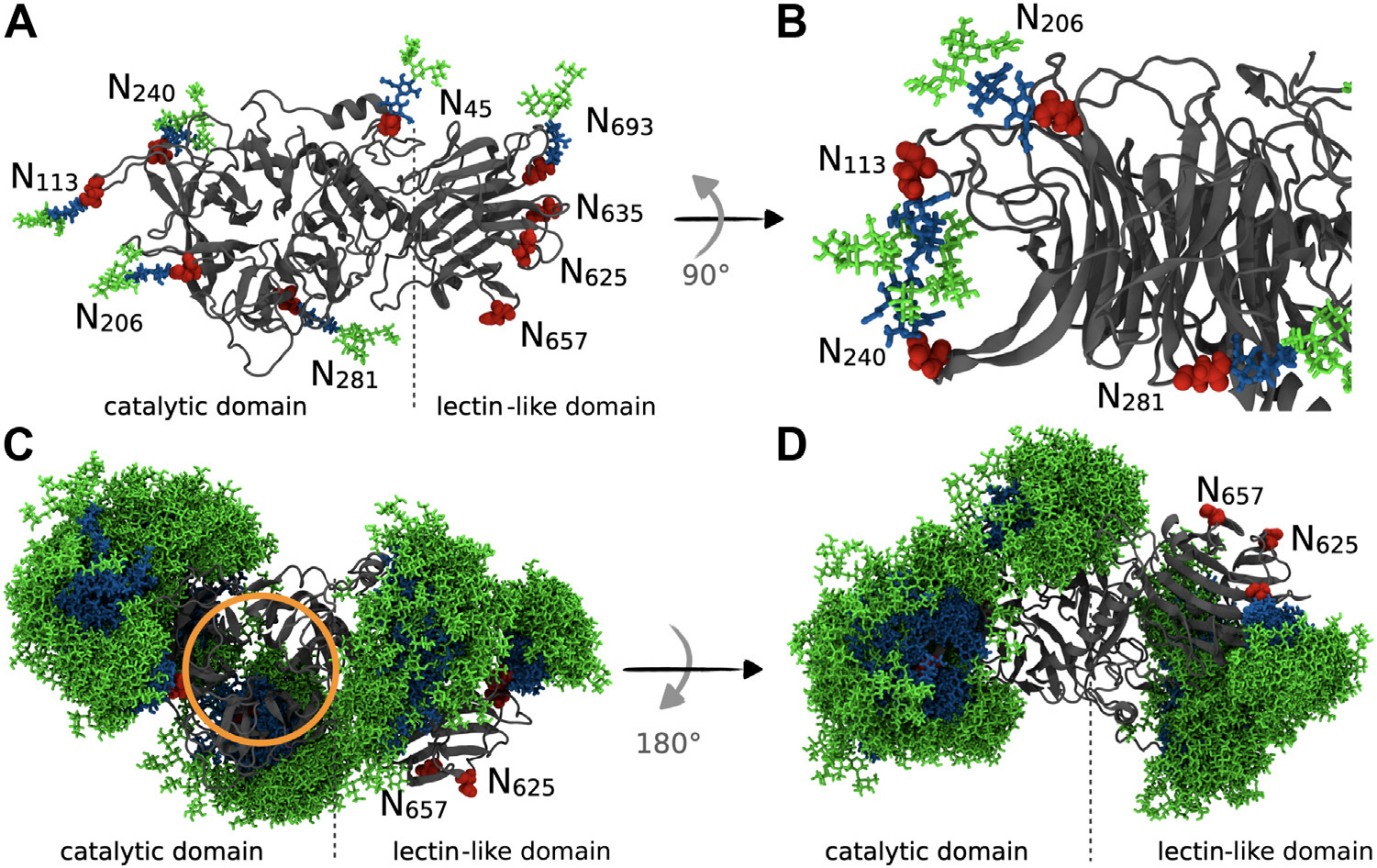
**Analysis of the dynamics of the *N*-glycan shield of TconTS1.***A*, atomistic model of TconTS1 with Man_5_GlcNAc_2_*N*-glycans (Man: *green*, GlcNAc: *blue*) at the asparagine residues (*red*) identified in MALDI-TOF MS experiments. *B*, interactions between N240 and N113 glycans mediated by hydrogen bonds observed during the MD simulations. *C*, overlay of all *N*-glycan positions recorded every 5 ns over a simulation time of 500 ns, with the protein backbone (*gray*) aligned in all frames and the active site indicated by an *orange circle*. *D*, same as *C*, with the protein rotated by 180°. The C-terminal SNAP-*Strep* is not shown in all structures. MD, molecular dynamics; MS, mass spectrometry; TconTS1, *Trypanosoma congolense* trans-sialidase 1.

At first, standard MD simulations were performed for TconTS1 (Fig. S7*A*) and H-TconTS1 (Fig. S7*B*) without bound substrates, in order to observe the dynamical behavior of the covalently linked *N*-glycans. Six of nine asparagine residues in the motif N-X-S/T are located at the tail of loop regions (Fig. 5*A*), which are mostly part of turns or coils framed by β-sheet regions. Results of our in-depth analysis can be found in Section S5 in Fig. S8. The terminal position and flexibility of these structural elements allow for large motion amplitudes and internal flexibility of the *N*-glycan trees. These movements enable interactions among glycans in structural proximity, for instance, intermolecular hydrogen bonds between the *N*-glycans at positions N113 and N240 (Fig. 5*B*), notwithstanding their distance in the protein sequence. Furthermore, an overlay of the averaged glycan distribution recorded every 5 ns during the simulation (Fig. 5, *C* and *D*) revealed a dense glycan coverage (shielding) of the protein, especially for the catalytic domain, except for the direct entrance to the active site (Fig. 5*C*).

#### Dynamics of conserved amino acids in the catalytic domain of substrate-free TconTS1

Interactions of *N*-glycans with highly conserved amino acids essential for the catalytic activity of TconTS1 (34, 51) were analyzed over the 500 ns MD trajectories. We especially focused on D150, E324, and Y438, known to be directly involved in catalysis, and on R126, R144, Y211, W212, R339, Y408, and R410, which are involved in substrate binding.

**Figure 6:**
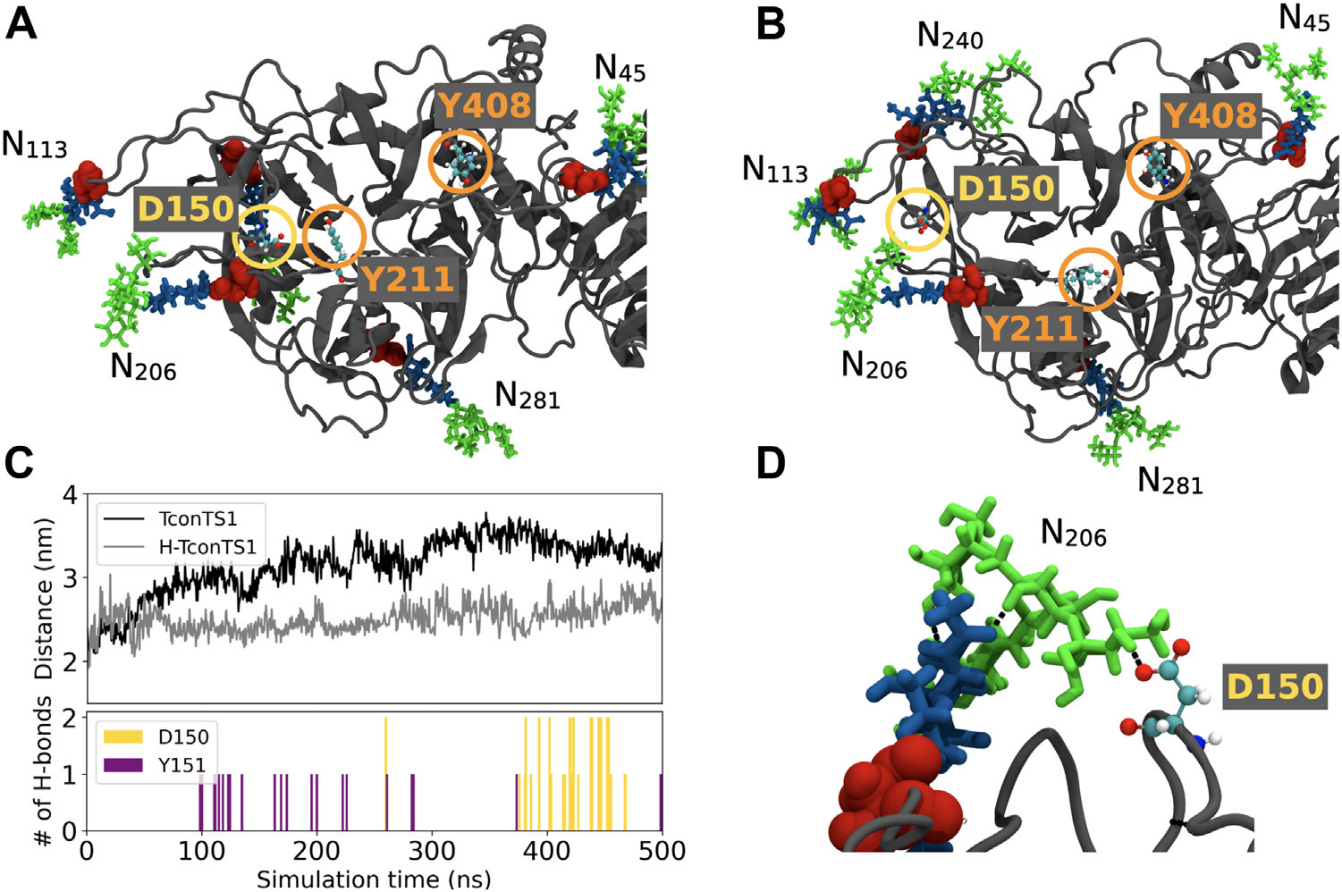
**Protein–glycan interactions drive active-site rearrangements observed in MD simulations of TconTS1 without substrate.***A*, amino acids of the catalytic site were in close proximity at the beginning of the simulation (snapshot at 100 ns). *B*, D150 moved out of the catalytic site and formed hydrogen bonds with glycan N206 until the end of the simulation (snapshot at 350 ns). *C*, time evolution of the distance between the center of D150 and the center of R410 for TconTS1 (*black*) and H-TconTS1 (*gray*) as well as numbers of hydrogen bonds for TconTS1 between glycan N206 and D150/Y151. For H-TconTS1, no hydrogen bonds were observed. *D*, detail of the hydrogen bonds (*black + dashed lines*) between D150 and glycan N206 at its terminal mannose branches. D150 is circled in *yellow*, and the ligand-binding residues Y211 and Y408 are circled in *orange* and represented in ball-and-stick with the following color code: oxygen (*red*), carbon (*cyan*), nitrogen (*blue*), and hydrogen (*white*). The underlying protein structure is represented in *cartoon* style in *gray* with asparagine residues of *N*-glycosylation sites labeled in *red spheres*. Glycan color code: Man (*green*) and GlcNAc (*blue*). H-TconTS1, hypoglycosylated TconTS1; MD, molecular dynamics; TconTS1, *Trypanosoma congolense* trans-sialidase 1.

In TconTS1, D150 was observed to shift from the interior of the active site (Fig. 6*A*) toward an exterior position (Fig. 6*B*), increasing its distance from R410, which was stationary within the active site (Fig. 6*C*). Interestingly, this shift seems to be stabilized by a hydrogen bond formed between D150 and the *N*-glycan at position N206 (Fig. 6, *C* and *D*). Further detailed analysis revealed that this process was initiated by hydrogen-bond formation between Y151 and the *N*-glycan at N206, already leading to a partial shift of D150 and making it more accessible to interact with the *N*-glycan (Fig. 6*C*). In contrast, for H-TconTS1, D150 was found to be by far less mobile (Fig. 6*C*).

**Figure 7:**
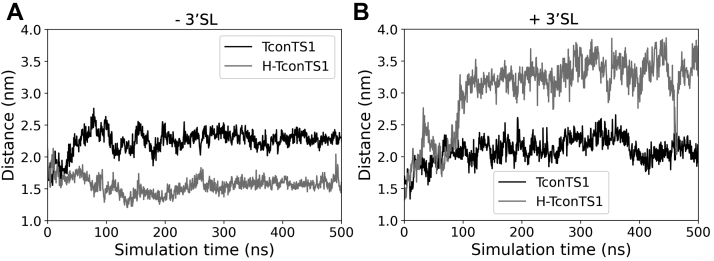
**Altered movement of lactose holder amino acids in EndoH**_**f**_**-treated H-TconTS1 compared with untreated TconTS1.** Distance in nanometer between the Cα of Y211 and Cα of Y408 for TconTS1 (*black*) and H-TconTS1 (*gray*) measured over time in nanoseconds. *A*, simulations without substrate. *B*, simulations with substrate. 3′SL, 3′-sialyllactose; EndoH_f_, endoglycosidase H; H-TconTS1, hypoglycosylated TconTS1; TconTS1, *Trypanosoma congolense* trans-sialidase 1.

For TcruTS, two amino acids, Y119 and W312, have been proposed to bind lactose and stabilize its position in the catalytic domain once a substrate is bound in the active site (52, 53). The relative orientation of Y119 and W312 can be described as an open or stacked conformation. Consistent with the results reported by Mitchell *et al.* (53), the corresponding amino acids, Y211 and Y408, in TconTS1 (Fig. 6, *A* and *B*, *orange circles*) exhibit a high flexibility and presented an open conformation with an average distance of 2.2 nm in the absence of a substrate (Fig. 7). For comparison, these two residues stayed in much closer contact in H-TconTS1, remaining at a distance of about 1.6 nm during our MD simulations (Fig. 7*A*).

#### Dynamics of conserved amino acids in the catalytic domain of substrate-bound TconTS1

**Figure 8:**
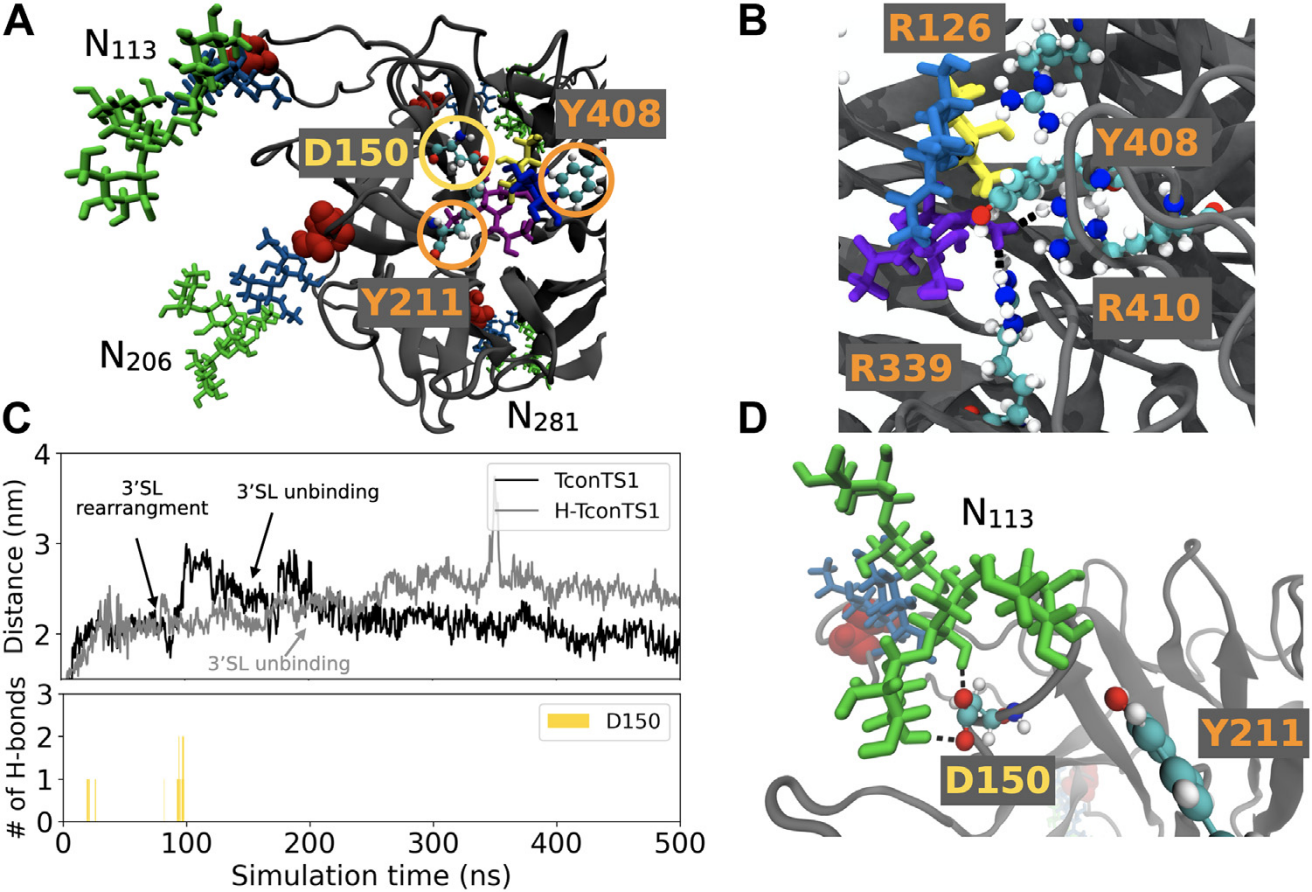
**MD simulations of TconTS1 with 3′SL bound to the active center revealed protein–glycan interactions.***A*, starting structure. *B*, snapshot of 3′SL in the binding pocket, forming hydrogen bonds (*black dashed lines*) to conserved arginine residues. *C*, distance between the center of D150 and the center of R410 for TconTS1 (*black*) and H-TconTS1 (*gray*). Number of hydrogen bonds between D150 and glycan N113 for TconTS1 over the course of the simulation. *D*, D150 interacts with glycan N113 *via* hydrogen-bond formation (*black dashed lines*) at its mannose branches (snapshot at 97 ns). Color code of the amino acids as for Figure 6. Glycan color code: Man (*green*), GlcNAc/Glc (*blue*), Gal (*yellow*), and Neu5Ac (*violet*). 3′SL, 3′-sialyllactose; H-TconTS1, hypoglycosylated TconTS1; MD, molecular dynamics; TconTS1, *Trypanosoma congolense* trans-sialidase 1.

As a next step, MD simulations of TconTS1 and H-TconTS1 were performed in complex with the substrate 3′SL. 3′SL was positioned in the binding site of TconTS1 in alignment with the crystal structure of the TcruTS–3′SL complex (Protein Data Bank [PDB] entry: 1S0I). In the starting structure, 3′SL is bound at the acceptor substrate-binding site between Y211 and Y408, and in close contact to both D150 and the well-conserved arginines, R339 and R410 (see Fig. 8, *A* and *B* for TconTS1).

As already seen in the substrate-free simulation, D150 of TconTS1 formed hydrogen bonds with mannose residues of an *N*-glycan. However, in this case, it was *N*-glycan at N113, which is also structurally in close proximity to the active site, and not the *N*-glycan at N206 (Fig. 8*D*). This interaction was observed after 20 ns, when D150 moved slightly off the catalytic site, becoming more accessible for interactions with the *N*-glycan (Fig. 8*C*). Following a structural rearrangement of 3′SL within the binding site after around 70 ns, D150 again interacted with *N*-glycan N113 and was dragged out of the binding site (Fig. 8*C*). In striking contrast, amino acids in H-TconTS1 known to be essential for the direct catalytic activity did not experience any interactions with residual GlcNAc residues, remaining after EndoH_f_ treatment.

In the presence of a substrate, different motions of Y211 and Y408 were observed with respect to the substrate-free protein. Namely, the distance between the two amino acids remained at about 2 nm in TconTS1 but increased from about 1.5 nm to more than 3 nm in H-TconTS1 in the presence of 3′SL (Fig. 7*B*).

## Discussion

In this study, we have focused on elucidating the potential influence of *N*-glycosylation on enzyme activity and stability of TconTS1 from the animal pathogen *T. congolense*. Therefore, recombinant TconTS1 was expressed in CHO Lec1 cells (34, 44). Hypoglycosylated TconTS1 samples were generated by EndoH_f_ treatment to investigate the lack of *N*-glycosylation on enzyme functions. Point mutations of single *N*-glycosylation sites have not been considered in this work, as a correct folding and function of TconTS1 was important to ensure comparable enzyme activities, and cotranslational *N*-glycosylation is known to influence protein folding. Our numerous experiments on site-directed glycosylation knockout using myelin-associated glycoprotein (Siglec-4, unpublished data) yielded largely misfolded proteins with loss of function, whereas after enzymatic deglycosylation of the wildtype protein, the function was retained. Thus, the approach of site-directed glycosylation knockout also has its limitations. Furthermore, for TconTS, the orders of magnitude of the lower specific activity of enzyme expressed in bacteria relative to that expressed by CHO Lec1 cells provide evidence for misfolding of the enzyme as a consequence of the absence of *N*-glycans (37).

Since TconTS1 exhibits nine putative *N*-glycosylation sites, a first assessment of which sites might be more important than others for enzyme function was desirable. Using qualitative MALDI-TOF MS analyses, we were able to demonstrate that at least seven of nine potential *N*-glycosylation sites in TconTS1 are conjugated with high-mannose type *N*-glycans, of which five are located in the catalytic domain. A heterogeneous *N*-glycosylation pattern at some sites was observed especially at N206 as also higher mannose structures (Man_6–8_GlcNAc_2_) and potentially also fucosylated *N*-glycans (Man_5_GlcNAc_2_Fuc) were detected in several, but not all, mass spectra. This heterogeneity might be explained by the lack of accessibility of the glycan for glycosidases, since *N*-glycan precursors are trimmed from Man_8_GlcNAc_2_ to Man_5_GlcNAc_2_ in the Golgi (54). This hypothesis is supported by our observation that EndoH_f_ was not able to remove all *N*-glycans from TconTS1 even after 16 h of incubation. In addition, our MD simulations revealed protein–glycan interactions between D150 and *N*-glycans at position N113 and N206 by hydrogen-bond formation. It was previously shown by biochemical *in vitro* assays and computational studies that such interactions decrease glycan accessibility, which might interfere with glycan trimming as well as with EndoH_f_-mediated removal of *N*-glycans (55). The potential presence of fucosylated *N*-glycans such as Man_5_GlcNAc_2_Fuc is a result of fucosyltransferase FUT8 activity, which was reported to act on Man_5_GlcNAc_2_ glycans (45, 56, 57). The detection of the sites N113, N206, N240, and N693 in either a glycosylated state or a nonglycosylated state is another form of heterogeneity already reported for other proteins (58).

The enzymatic removal of high-mannose type *N*-glycans did not disrupt the secondary structure of TconTS1. First, CD spectra of TconTS1 and H-TconTS1 did not reveal any detectable differences in secondary structure elements. Second, a similar midpoint of unfolding transition was determined for both enzymes in temperature-ramping experiments, suggesting no differences in stability. Similar results were found in single-site *N*-glycosylation mutants of the infectious bronchitis virus spike protein by means of CD experiments (59). The observed thermal stability of TconTS1 might be explained by the high β-sheet content of the protein as well as by an extended interface between the catalytic and lectin-like domain stabilized by salt bridges and a well-structured hydrogen-bond network, making unfolding rather unlikely (37). Although *N*-glycans do not seem to influence the stability of TconTS1 after successful expression, *N*-glycans are known to be required for proper folding of *N*-glycosylated proteins regulated by the calnexin–calreticulin cycle in the endoplasmic reticulum (60). Whether this is true also for TconTS1 still needs to be investigated, although enzymatic activity of bacterially expressed TconTS provides evidence for glycosylation-dependent misfolding of the enzyme (37).

A clear influence of *N*-glycosylation on enzymatic activity, however, was observed. Lactose affinity of H-TconTS1 was decreased by a factor of 1.6 to 5 relative to TconTS1. However, the *V*_max_ value was the same for both enzymes, which corroborates the structural integrity of H-TconTS1. Differences between previously reported *V*_max_ values for TconTS1 (4.1 ± 0.1 μmol 3′SL/[min × mg enzyme]) (34) are most likely because of different enzyme preparations. It could be further argued that the glycosylated TconTS1 control in this study was incubated for 16 h at 37 °C before the assays, equally to the EndoH_f_-treated enzyme (see the Experimental procedures section) and that this has caused a lower *V*_max_. However, this is unlikely, since previous unpublished work in our laboratory has shown that prolonged incubation (even for weeks) at 37 °C does not lead to loss of activity (39). Furthermore, the CD experiments of this study provided evidence for a good heat stability of TconTS1.

Haynes *et al.* (21) studied the influence of *N*-glycosylation on TvivTS1 (*Trypanosoma vivax* trans-sialidase 1) and did not observe an effect on enzyme activity. The same applies to investigations of a mutated variant of TranSA, which expresses TS activity (22). However, these studies did not determine the *K*_*M*_ values for the substrates used in enzyme reactions. Another study of TranSA, in which the sialidase activity was investigated, did not observe strong effects on *K*_*M*_ when recombinant proteins were expressed in *Escherichia coli* and compared with the native enzymes isolated from trypanosomes (42). However, sialidase activity of TS is determined in the absence of a Sia acceptor substrate such as lactose. For TcruTS, enzymes expressed in *E. coli* still showed transfer activity although to a lesser extent than observed for the native protein, which might be a result of the absence of *N*-glycans and/or incorrect protein folding (40).

Molecular insight into the putative effect of *N*-glycosylation on TconTS1 at the atomic level was obtained in a series of MD simulations. The *N*-glycans, especially in TconTS1 catalytic domain, were observed to form a highly dynamical “shield” enclosing the enzyme, while leaving the entrance to the catalytic center open for substrate binding.

In several simulations of TconTS1, both in the absence and presence of bound 3′SL, D150 forms hydrogen bonds with *N*-glycans at positions N206 or N113. Thus, these glycans may impact the proton-donation ability of this aspartate in the enzymatic transfer of Sia, a crucial contribution to substrate conversion (17, 51). Interestingly, these are conserved *N*-glycosylation sites among TS or sialidases from different species, despite variations in amino acid sequences and distributions of other *N*-glycosylation sites. In particular, N206 is conserved not only in all TS variants of *T. congolense* as already described by Waespy *et al.* (25) but also in enzymes from *T. cruzi*, *T. brucei*, *T. vivax*, and in the sialidase from *T. rangeli* as revealed by our amino acid sequence alignment (Fig. S3). In addition, N113 is also conserved in TconTS, TvivTS, and TranSA.

Along this line, we propose that there might be a common mechanism of TS activity mediated by *N*-glycan interactions with amino acids of the active site, in particular with D150 or its equivalents in other TS. The short time scale and lack of *N*-glycans in previously performed MD simulations probably prevented the exploration of the D150 structural shift (52, 53, 61, 62). Also, the arrangement of Y211 and Y408 was expected to participate in binding of the acceptor lactose and appears to be influenced by the presence of *N*-glycans. In the TconTS1 model, the distance between these amino acids (about 2 nm) was not changed in the presence of the donor substrate 3′SL, whereas in the corresponding H-TconTS1 model, the two amino acids, Y211 and Y408, increased from a close proximity (1.5 nm) to twice the distance (above 3 nm) if 3′SL was present in the active site. These observations are in agreement with the notion that *N*-glycans modulate the fine tuning of critical amino acid side-chain arrangements at the catalytic site of TconTS1, which might facilitate the initial binding of substrates and lead to a higher substrate-binding affinity in line with our kinetic data.

It has to be noted that the heterogeneity of *N*-glycosylation at different sites (Table 1) was not considered in our MD simulations at this stage. Investigating the influence of individual *N*-glycans or their specific structures on modulating catalytic activity represents an elaborate task and should be studied in more detail in the future.

Intramolecular glycan–protein interactions are often observed (63) and have been suggested to regulate the conformation of proteins and their substrate-binding ability. For example, it has been demonstrated that *N*-glycosylation affects substrate affinity using a recombinant exoinulinase from *Kluyveromyces cicerisporus* (64). This observation can be confirmed by our study for TS enzymes and highlights the importance of protein glycosylation on substrate binding. Furthermore, our study underlines the importance of explicitly including *N*-glycans in MD simulations because of their crucial functional role, as was shown for the spike protein of severe acute respiratory syndrome coronavirus 2 (65).

To the best of our knowledge, this is the first time that interactions of the *N*-glycan shield with amino acids of the catalytically active site of an enzyme were observed to modulate enzymatic activity. As *N*-glycosylation sites are partially conserved among the TS enzyme family, the here-observed protein–glycan interactions may also occur in TS of other trypanosome species.

## Experimental procedures

Chemicals and reagents used in this study were of cell-culture and analytical grade and purchased from Sigma–Aldrich/Merck KGaA or Carl Roth if not stated otherwise.

### Expression of TconTS1 in CHO Lec1 cells

Recombinant TconTS1 was produced in CHO Lec1 cells as already described (34). In brief, a modified pDEF vector was used for stable transfection comprising a transin sequence for protein secretion followed by the TconTS1-encoding sequence excluding signal peptide and GPI anchor sequence, a C3 protease cleavage site, and a C-terminal SNAP and *Strep* tag. Monoclonal cells were grown in serum-free CHO medium (Bio&SELL) or Excell medium supplemented with 50 μg/ml gentamicin sulphate (Lonza BioWhittaker) at 37 °C and 5% CO_2_.

### Purification of TconTS1 from cell culture supernatant

Cell culture supernatant comprising the recombinant protein was harvested every second day and stabilized with 10 mM Tris–HCl, pH 8.0, 10 mM EDTA, 10 mM ascorbic acid, and 0.02% sodium azide. Ultracentrifugation was performed at 7800 rcf for 15 min followed by 40,000 rcf for 45 min at 4 °C. The clear supernatant was microfiltered (0.22 μm, polyethersulfone) and concentrated to 50 ml using a Sartorius Vivacell 250 PES Centrifugal Concentrator (Sartorius) with a molecular weight cutoff of 100 kDa and a pressure of 4 bar. Buffer was exchanged five times with 200 ml of 100 mM Tris–HCl (pH 8.0), 150 mM NaCl, 1 mM EDTA, and concentrated to a final volume of 10 ml. The concentrate was centrifuged at 21,000 rcf for 30 min, and recombinant protein was purified from the supernatant with *Strep*-Tactin sepharose (IBA) according to the manufacturer’s protocol. Elution fractions containing TconTS1 were pooled, and buffer was exchanged four times with 5 ml of 10 mM potassium phosphate buffer (pH 7.4) using a Vivaspin 6 Centrifugal Concentrator (Sartorius) at 2000 rcf and 4 °C for 20 min. Protein samples were stored at 4 °C. TconTS1 concentrations were determined with a Pierce BCA Protein Assay kit (Thermo Fisher Scientific) using bovine serum albumin as standard.

### EndoH_f_ treatment of TconTS1

About 2 mg of TconTS1 were incubated with 40,000 units of EndoH_f_ (New England Biolabs) for 16 h at 37 °C in 2.0 ml 10 mM phosphate buffer (pH 7.4). The EndoH_f_-treated enzyme is termed H-TconTS1. A TconTS1 control without EndoH_f_ addition was performed in parallel (TconTS1). The H-TconTS1 sample of replicate 1 was purified again using *Strep*-Tactin sepharose to remove free glycans and EndoH_f_, and buffer was exchanged to 10 mM phosphate buffer (pH 7.4) as already described. In contrast, the H-TconTS1 sample of replicate 2 was purified by three chromatography steps: (i) a PD-10 desalting column (Cytiva) to remove free glycans, (ii) by AffiSep ConA adsorbent (Galab) to remove remaining proteins with high-mannose type *N*-glycans, and (iii) finally treated by an amylose affinity purification (New England Biolabs) to remove EndoH_f_ by its fused maltose-binding protein. All chromatography steps have been performed according to the manufacturer’s protocols. Finally, the buffer was exchanged again to 10 mM phosphate buffer (pH 7.4).

### SDS-PAGE, Western blot, and ConA lectin blot analysis

Protein samples were separated *via* SDS-PAGE and either stained with PageBlue Protein Staining Solution (Thermo Fisher Scientific) or used for Western blot or ConA lectin blot analysis. For Western blots, a polyclonal rabbit anti-*Strep* (IBA) and a polyclonal peroxidase-conjugated donkey anti-rabbit antibody (Jackson ImmunoResearch) were used as a primary and secondary antibody, respectively. For ConA lectin blots, high-mannose *N*-glycosylated proteins were detected employing ConA-biotin (Galab) and the VECTASTAIN ABC-HRP Kit (Vector Laboratories).

### MALDI-TOF MS analysis of TconTS1 *N*-glycosylation sites

In-solution digestion of glycosylated and hypoglycosylated TconTS1 was performed with trypsin and chymotrypsin (Promega) to analyze peptides with MALDI-TOF MS. Protein solutions with a final concentration of 0.1 mg/ml were prepared in 50 mM NH_4_HCO_3_ buffer (pH 7.8). Cysteine bonds were reduced with 1.4 mM DTT for 30 min at 50 °C, and the protein was denatured for 10 min at 95 °C. After a short cooling step on ice, cysteine residues were alkylated with iodoacetamide at a final concentration of 3.2 mM for 30 min at 37 °C and protected from light. Protein samples were digested with trypsin or chymotrypsin overnight at 37 °C at protein:protease ratios of 25 to 100:1. Two negative controls were prepared accordingly, either lacking the protein TconTS1 (N1) or the protease (N2).

Samples were spotted directly on the target (Bruker Daltonics), or glycopeptides were further purified with ConA beads as described under “Glycopeptide purification of protease-digested TconTS1 using ConA beads” section. For spotting, 1 μl protein solution was directly mixed with 1 μl α-cyano-4-hydroxycinnamic acid matrix solution (40 mM in 50% acetonitrile/0.1% TFA) on the target. Samples were measured in the positive reflector mode using the MALDI-TOF autoflex speed (Bruker Daltonics), which was calibrated with the peptide calibration standard II (Bruker Daltonics). Detailed instrument settings can be found in Table S1. The peak assignment procedure and validation criteria are summarized in Section S3 of the supporting information.

Since our MALDI-TOF MS device does not reveal single protein-resolved *N*-glycosylation pattern, no in-depth glycoproteome analysis was performed. A comparison of spectra generated from H-TconTS1 peptides of both replicates 1 and 2 has not been performed. Glycoproteome analyses were only carried out with replicate 1. Replicate 2 originated from an initial study focusing mainly on enzyme kinetics. Quantities of replicate 2 were not sufficient for a comprehensive glycoproteome analysis.

### Glycopeptide purification of protease-digested TconTS1 using ConA beads

To concentrate glycopeptides from protease-digested TconTS1, samples were incubated with AffiSep ConA adsorbent (Galab). The adsorbent was equilibrated in 1× binding buffer (50 mM Tris–HCl [pH 7.4], 150 mM NaCl, 1 mM CaCl_2_, 1 mM MgCl_2_, and 1 mM MnCl_2_) and was added to trypsin-digested and heat-inactivated protein samples in a ratio of 1 μl adsorbent/2.5 μg protein. About 5× binding buffer of appropriate volumes was supplied to samples to yield a final concentration of 1×. Samples were rotated overnight at 8 rpm and 4 °C and centrifuged for 30 s at 130 rcf. The supernatant was discarded, and the adsorbent was washed thrice with 50 mM NH_4_HCO_3_ buffer (pH 7.8) in binding buffer and at the same centrifugation conditions. After the last centrifugation step, the adsorbent was resuspended in 10 to 50 μl of NH_4_HCO_3_ buffer (pH 7.8). Elution of glycopeptides was performed at 95 °C for 10 min. Samples were centrifuged at 4300 rcf for 30 s. The supernatant was used for MALDI-TOF MS analysis. Negative controls were prepared in parallel with identical components apart from the trypsin-digested TconTS1. Peaks resulting from negative controls were excluded for data analysis.

### TS activity assay

TS activity assays were executed as previously described (34, 39). Fetuin and lactose served as Sia donor and acceptor, respectively. Reactions were carried out in 50 μl of 10 mM potassium phosphate buffer, pH 7.4, containing 100 μg dialyzed fetuin (corresponding to 600 μM bound Neu5Ac), varying concentrations of lactose (0.01–5 mM), and 50 ng hypoglycosylated TconTS1 or the corresponding control. Samples were incubated at 37 °C for 30 min, and the reaction was terminated with 200 μl ice-cold acetone. After protein precipitation overnight at −20 °C, samples were centrifuged (20,000 rcf, 30 min, 4 °C), lyophilized, and resuspended in 125 μl water. Transfer activity was measured as 3′SL implementing the HPAEC–PAD system ICS-5000+ (Dionex/Thermo Fisher Scientific). About 25 μl of the sample was applied to a CarboPac100 analytical column (250 × 2 mm, 8.5 μm; Thermo Fisher Scientific) equipped with a guard column (50 × 2 mm; Thermo Fisher Scientific). Chromatography was performed at isocratic conditions with 100 mM NaOH and 100 mM NaOAc for 12 min followed by a wash step with 100 mM NaOH and 500 mM NaOAc for 5 min and an equilibration step for 8 min to previous conditions. Production of 3′SL was quantified with a purchased 3′SL standard (Carbosynth). Data acquisition and evaluation was performed with the Dionex software Chromeleon 7.2 SR5. Parameters of the Michaelis–Menten equation, *K*_*M*_ and *V*_max_, were calculated with the curve fit model of SigmaPlot11 (Systat Software GmbH; http://www.systat.de/SigmaPlot11_Produktseite.html).

### CD experiments

CD experiments were performed with purified recombinant TconTS1 in its glycosylated and hypoglycosylated forms dissolved in 10 mM phosphate buffer (pH 7.4). The Applied Photophysics Chirascan spectrometer (Applied Photophysics Limited) with the Pro-Data Chirascan software (version 4.2.22) was used to record and evaluate CD spectra. For each sample, at least three repetitive scans were performed over a standard wavelength range of 190 to 250 nm with intervals of 1 nm. Throughout the experiments, Suprasil quartz cells (Hellma UK Ltd) were used with a path length of 0.2 mm. Baseline scans were performed with 10 mM phosphate buffer (pH 7.4) only, in the respective cuvette. Regarding data processing, the baseline was subtracted from recorded spectra, and repetitive scans were averaged before a Savitsky–Golay smoothing filter with smoothing windows of three data points was applied. Spectra have been recorded in raw ellipticity (Θ) and were further converted to mean residue ellipticity.

$$\Theta_{MRE}=\frac{\Theta }{cln}$$

where *c* is the protein concentration, *l* the quartz cuvette path length, and *n* the number of amino acids in the protein sequence. To be consistent with the parameters of the enzyme assay, constant-temperature measurements were performed at 35 °C to check for a general influence of glycans on the protein structure.

To estimate the secondary structure components of the measured sample, CD spectra were analyzed using the BeStSel Web server (66, 67). Temperature-ramping experiments were performed following the suggestions (68) in order to analyze protein stability, unfolding intermediates, and the midpoint of the unfolding transition (*T*_M_). In detail, protein samples were heated from 20 °C up to 95 °C with 5 °C temperature steps employing the stepped ramp mode. After 5 min of equilibration time at the respective temperature, at least three spectra were recorded and averaged. The *T*_M_ was calculated from the fraction of protein folded at any temperature (α) defined as:

α=(ΘT−ΘU)/(ΘF−ΘU),

where ΘT is the ellipticity at any temperature, ΘU is the ellipticity at the unfolded state, and ΘF at the folded state. *T*_M_ is defined as the temperature at which α = 0.5 (68) and also referred to as the melting temperature. In order to calculate α, we chose 195 nm as the wavelength to plot recorded $\Theta_{MRE}$ values against the temperature. Afterward, the calculated α values were again plotted with reference to temperature, and a sigmoid curve was used for fitting to obtain a precise TM value. As we did not observe a complete unfolding of TconTS1 in any temperature-ramping experiment, ΘU is defined as the average ellipticity of the two highest temperatures. Therefore, ΘF was set as the average ellipticity that was recorded for the two lowest temperatures.

### Atomistic model of glycosylated and deglycosylated TconTS1

Structures of TS from *T. congolense* have not been resolved so far by experimental techniques. Therefore, an atomistic structure of TconTS1 was modeled by the I-TASSER web server for protein structure and function predictions (69, 70) based on the recombinant sequence without the transin signal (Fig. S1; amino acids 23–951). However, the numbering of amino acids is in correspondence with the native sequence, as depicted in Fig. S1*B* (34). As TconTS1 was modeled with the engineered SNAP-*Strep* for consistency and better comparison with experimental data, restraints were used to achieve proper folding of this structural part. In detail, a secondary structure restraint as well as a structure template for the SNAP-*Strep* region, generated by I-TASSER beforehand using only the SNAP-*Strep* sequence, was employed. Major templates employed by the threading algorithm were TranSA (PDB entry: 2AGS and 2A75) as well as TcruTS (PDB entry: 1MS9). Analysis and validation of homology model construction is further outlined in Section S5 of the supporting information.

### Construction of the simulation cell

The freely accessible CHARMM-GUI Glycan Modeler (www.charmm-gui.org) was employed to form a disulfide bond between residues C493 and C503. For TconTS1, Man_5_GlcNAc_2_ *N*-glycans were included at positions N45, N113, N206, N240, N281, and N693 with CHARMM-GUI (71, 72, 73, 74, 75, 76) as identified with MALDI-TOF MS. Man_5_GlcNAc_2_ was chosen for all sites, as it represents the simplest and most often found *N*-glycan in CHO Lec1 cells (45). N657 was not glycosylated, although found in our MALDI experiments, because model building was already completed at the time this glycan was found. H-TconTS1 was generated by the addition of one GlcNAc residue *via* a covalent linkage at positions N45, N113, N206, N240, N281, and N693. Atomistic structures of generated models are also described in Section S5 of the supporting information (Fig. S7). The simulation box was constructed with CHARMM-GUI, filling it with water molecules to obtain a distance of 15 Å between the protein and box edge. About 22 K^+^ ions have been added for charge neutralization.

### MD simulation

All MD simulations were performed with the GROMACS 2018 version (77), using the CHARMM36m (78) force field for proteins, *N*-glycans, and 3′SL (79, 80) in combination with the TIP3P water model. The leap-frog algorithm was used as an integrator, and the LINCS algorithm (81) was employed to constrain bonds connected to hydrogen atoms. Temperature coupling was performed with velocity rescaling using a τ parameter of 0.1 ps (82). The verlet cutoff scheme (83) was employed for van der Waals parameters using particle mesh Ewald and the standardized parameters suggested for CHARMM36 in the GROMACS manual version 2019.

Energy minimizations of water and ions (with restrained proteins) were performed using the steepest descent algorithm with a tolerance of 1000 kJ mol^−1^ nm^−1^. Equilibration of water (with restrained proteins) was done in an NVT and an NPT ensemble for 1 ns, respectively. It followed the energy minimization of the proteins (with restrained water and ions) under the same conditions as before. Finally, unrestrained equilibrations were performed under NVT and NPT for 1 ns each. The production runs were performed for 500 ns in the NVT ensemble at 310.15 K, writing coordinates to file every 10 ps. A time step of 2 fs was set for all parts of the simulations if not mentioned otherwise. The systems were analyzed and visualized every 500 ps using VMD (http://www.ks.uiuc.edu/Research/vmd/), the open-source community-developed PLUMED library version 2.6 and Python version 3.7 (84, 85, 86, 87, 88).

### Construction of TconTS1 in complex with 3′SL

3′SL was chosen as a substrate since its composition is similar to the typical terminal branches of complex type *N*-glycans and was already used in previous enzyme assays (9). To ensure a correct binding pose of the ligand in the catalytic site, the homology-modeled TconTS1 structure was aligned to the PDB structure of TcruTS (PDB entry: 1S0I) in complex with 3′SL by VMD. The position of 3′SL was copied to the TconTS structure, and the ligand–protein complex was subjected to CHARMM-GUI for further processing, as described under “Construction of the simulation cell” section. Further minimization and equilibration steps were performed as described under the “MD simulation” section, using a time step of 1 fs or 2 fs to resolve steric clashes. Production runs were performed as mentioned previously.

## Data availability

The underlying data of this study, such as raw spectra of MALDI-TOF MS and CD experiments as well as structure and trajectory files of MD simulations, are made available under https://doi.org/10.5281/zenodo.6102786. Plumed files used in this study are stored in the Plumed-NEST repository (21.050).

## Supporting information

This article contains supporting information (34, 39, 45, 51, 66, 67, 69, 70, 89, 90, 91, 92, 93, 94, 95).

## Conflict of interest

The authors declare that they have no conflicts of interest with the contents of this article.
