# Supplementary material for: N-glycosylation modulates enzymatic activity of Trypanosoma congolense trans-sialidase
Source: J Biol Chem. 2022 Aug 20;298(10):102403. doi: 10.1016/j.jbc.2022.102403 (PMC9493392; doi:10.1016/j.jbc.2022.102403)
Supplement: Supplemental Table S1 [file mmc1.docx]

| **Parameter** | **Setting** |
| --- | --- |
| Mass range | 500 – 4500/6000 |
| Smartbeam Parameter Set | Medium |
| Frequency | 500 Hz |
| Sample rate | 4 Gs/s |
| Baseline offset adjustment | 1.2 % |
| Analog offset | 51 mV |
| Ion source 1 | 19.11 kV |
| Ion source 2 | 16.91 kV |
| Lens | 8.69 |
| Reflector 1 | 20.98 kV |
| Reflector 2 | 9.44 kV |
| Pulsed ion extraction | 130 ns |
| Matrix suppression mode (up to) | Deflection (500) |
| Polarity | Positive |
| Detector gain voltage | 1700 V |
