# Supplementary material for: N-glycosylation modulates enzymatic activity of Trypanosoma congolense trans-sialidase
Source: J Biol Chem. 2022 Aug 20;298(10):102403. doi: 10.1016/j.jbc.2022.102403 (PMC9493392; doi:10.1016/j.jbc.2022.102403)
Supplement: Supplemental Table S3 [file mmc3.docx]

|  | TconTS1 | H-TconTS1 |
| --- | --- | --- |
| α-helix | 13.5 ± 0.2 | 13.2 ± 0.5 |
| β-sheet (antiparallel) | 37.1 ± 2.1 | 36.4 ± 2.5 |
| β-sheet (antiparallel) | 0 | 0 |
| turn | 11.3 ± 0.1 | 11.2 ± 0.0 |
| others | 38.2 ± 1.9 | 39.2 ± 2.0 |
