# Supplementary material for: N-glycosylation modulates enzymatic activity of Trypanosoma congolense trans-sialidase
Source: J Biol Chem. 2022 Aug 20;298(10):102403. doi: 10.1016/j.jbc.2022.102403 (PMC9493392; doi:10.1016/j.jbc.2022.102403)
Supplement: Supplemental Figure S1 [file mmc4.pdf]

A

1 10 20 30 40 50 60 70 80 90 100
MKGLPVLLWLCTAVCSSYPLHGSEEDAGMETSQCCDHMHATAAVGTTHQALLWGSKWALRNKTTPKDGEVWWSNPQPGWKEVYDDEWEEWFMEQKGPTGV

110 120 130 140 150 160 170 180 190 200
NGVRTEWYRRMKDGYILVGGPKLNSPDMNSTGTTMRTVHSYRIPSIVEVGGVLMCVGDARYITSTDYFFTDTVAAYSTDGGRTWKREVIIPNGRVDAHYS

210 220 230 240 250 260 270 280 290 300
RVVDPTVVAKGNNIYVLVGRYNVTRGYWHNKNNRAGVADWEPFVYKGTVNVGTKDNATDVSISWERTALKSLYNFPVSGSPGTQFLGGAGGGVVTSNGTI

310 320 330 340 350 360 370 380 390 400
VLPVQARNKANRVVSMILYSADDGKSWHFGKGEAGVGTSEAALTEWDGKLLISARSDGGQGYRMIFESSDLGATWKEMLNSISRVIGNSPGRSGPGSSSG

410 420 430 440 450 460 470 480 490 500
FITVTVEGVPVMLLTHPKNLKGSYYRDRLQMWMTDGNRMWHVGQVSEGDDNSAYSSLLYTPDGVLYCLHEQNIDEVYSLHLVRLVDELKSIKSTALVWKA

510 520 530 540 550 560 570 580 590 600
QDELLLGNCLPGDKYDPGCDGIPTAGLAGLLVGPLTEKTWPDAYRCVNAATSGAVSTAEGVRLDVGGGGHVVWPVSEQGQDQRYYFTNSEFTLAVTVRFD

610 620 630 640 650 660 670 680 690 700
EMPQGELPLLGFVNREGKVKKILKVSLSGVEWLLAYGNEYNSTAAEPLDVNESHQVVLALHDGIVSLHVDGGNTTATVSVRVASPAELLNIHHLFVGTPV

710 720 730 740 750 760 770 780 790 800
DGGAKEHANITVSNVLVYNRPLRGVELLGLFANRGRIRVPGSDGSLEVLFQGPMDKDCEMKRTTLDSPLGKLELSGCEQGLHEIKLLGKGTSAADAVEVP

810 820 830 840 850 860 870 880 890 900
APAAVLGGPEPLMQATAWLNAYFHQPEAIEEFPVPALHHPVFQQESFTRQVLWKLLKVVKFGEVISYQQLAALAGNPAATAAVKTALSGNPVPILIPCHR

910 920 930 940 949
VVSSSGAVGGYEGGLAVKEWLLAHEGHRLGKPGLGEFDIGTWSHPQFEK

B

1 10 20 30 40 50 60 70 80 90 100
MWPVNCYALLALVVAGQCCDHMHATAAVGTTHQALLWGSKWALRNKTTPKDGEVWWSNPQPGWKEVYDDEWEEWFMEQKGPTGVNGVRTEWYRRMKDGYI

110 120 130 140 150 160 170 180 190 200
LVGGPKLNSPDMNSTGTTMRTVHSYRIPSIVEVGGVLMCVGDARYITSTDYFFTDTVAAYSTDGGRTWKREVIIPNGRVDAHYSRVVDPTVVAKGNNIYV

210 220 230 240 250 260 270 280 290 300
LVGRYNVTRGYWHNKNNRAGVADWEPFVYKGTVNVGTKDNATDVSISWERTALKSLYNFPVSGSPGTQFLGGAGGGVVTSNGTIVLPVQARNKANRVVSM

310 320 330 340 350 360 370 380 390 400
ILYSADDGKSWHFGKGEAGVGTSEAALTEWDGKLLISARSDGGQGYRMIFESSDLGATWKEMLNSISRVIGNSPGRSGPGSSSGFITVTVEGVPVMLLTH

410 420 430 440 450 460 470 480 490 500
PKNLKGSYYRDRLQMWMTDGNRMWHVGQVSEGDDNSAYSSLLYTPDGVLYCLHEQNIDEVYSLHLVRLVDELKSIKSTALVWKAQDELLLGNCLPGDKYD

510 520 530 540 550 560 570 580 590 600
PGCDGIPTAGLAGLLVGPLTEKTWPDAYRCVNAATSGAVSTAEGVRLDVGGGGHVVWPVSEQGQDQRYYFTNSEFTLAVTVRFDEMPQGELPLLGFVNRE

610 620 630 640 650 660 670 680 690 700
GKVKKILKVSLSGVEWLLAYGNEYNSTAAEPLDVNESHQVVLALHDGIVSLHVDGGNTTATVSVRVASPAELLNIHHLFVGTPVDGGAKEHANITVSNVL

710 720 730 740 750
VYNRPLRGVELLGLFANRGRIRVPGSDNGVLSGGCLSLCYLLLLVHVLMF
