# Supplementary figures and images for: N-glycosylation modulates enzymatic activity of Trypanosoma congolense trans-sialidase
Source: J Biol Chem. 2022 Aug 20;298(10):102403. doi: 10.1016/j.jbc.2022.102403 (PMC9493392; doi:10.1016/j.jbc.2022.102403)

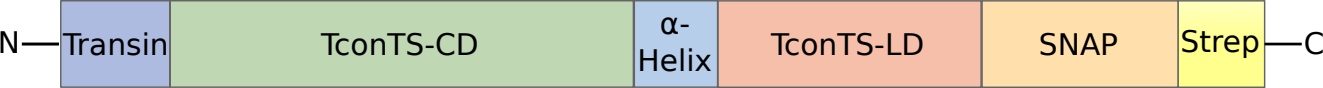

Supplement: Supplemental Figure S2 [file mmc5.pdf]

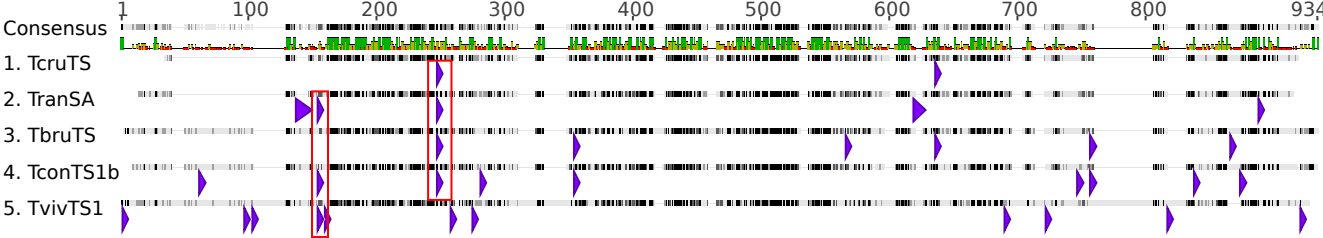

Supplement: Supplemental Figure S3 [file mmc6.pdf]

**A**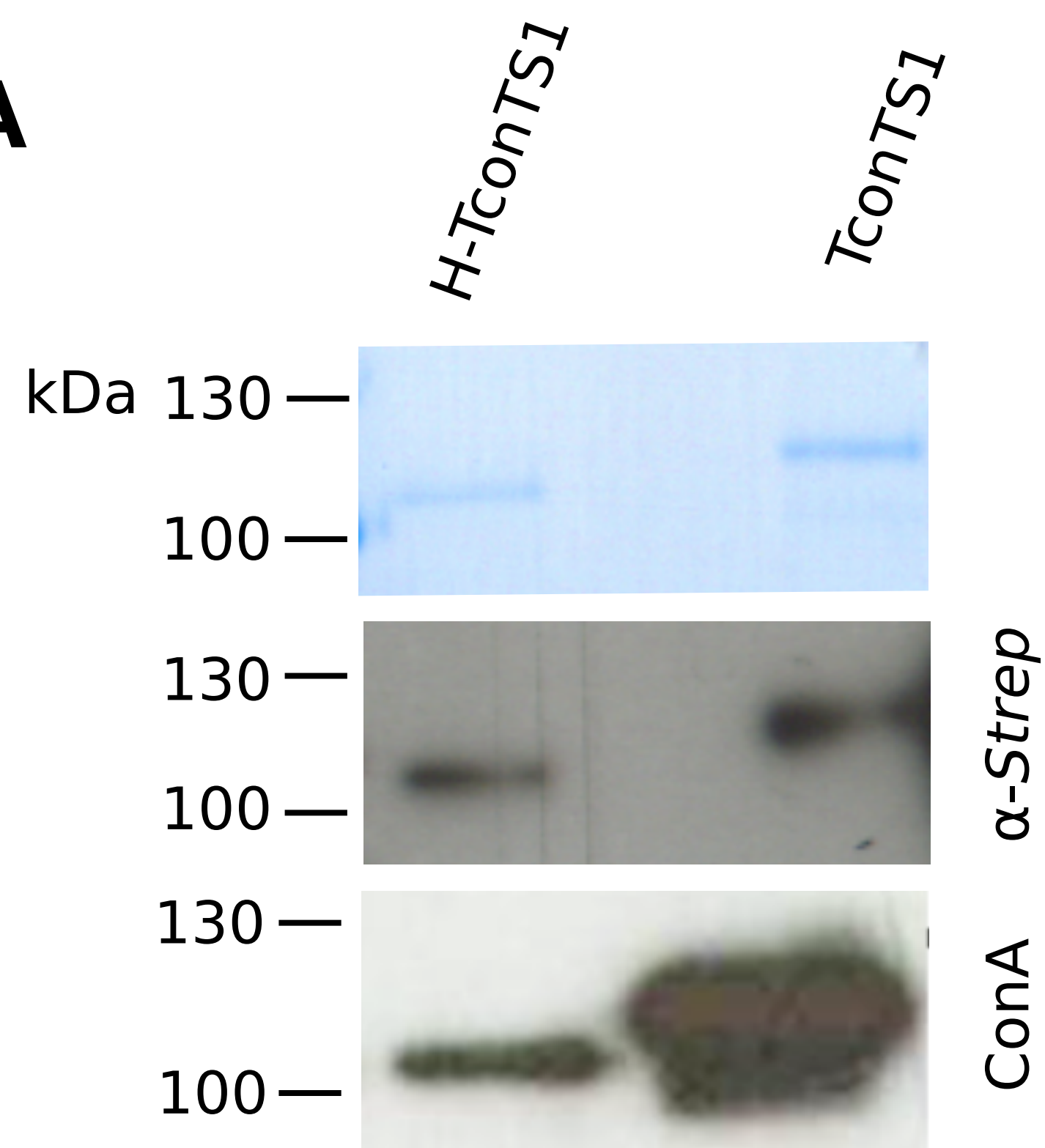**B**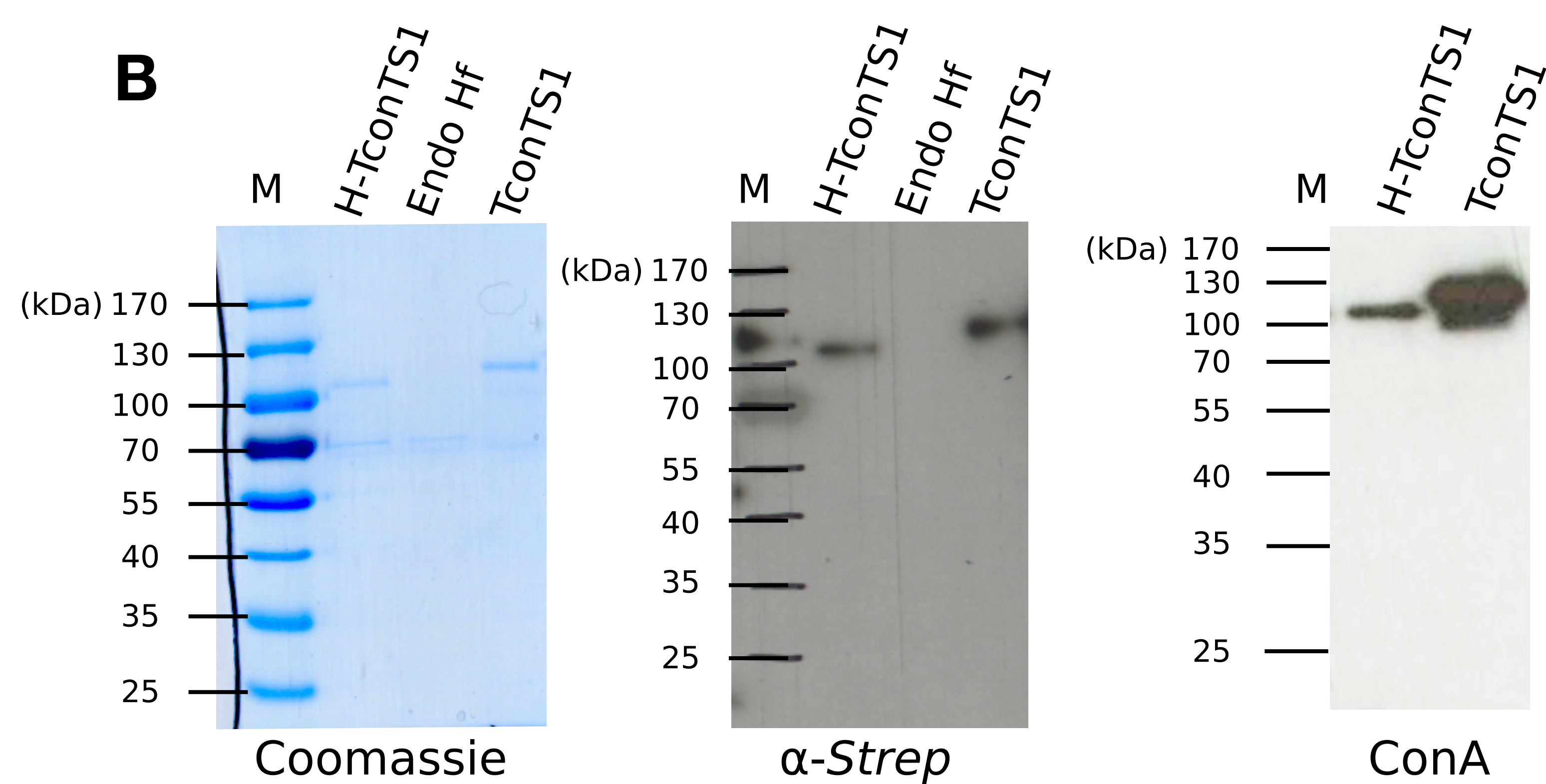**C**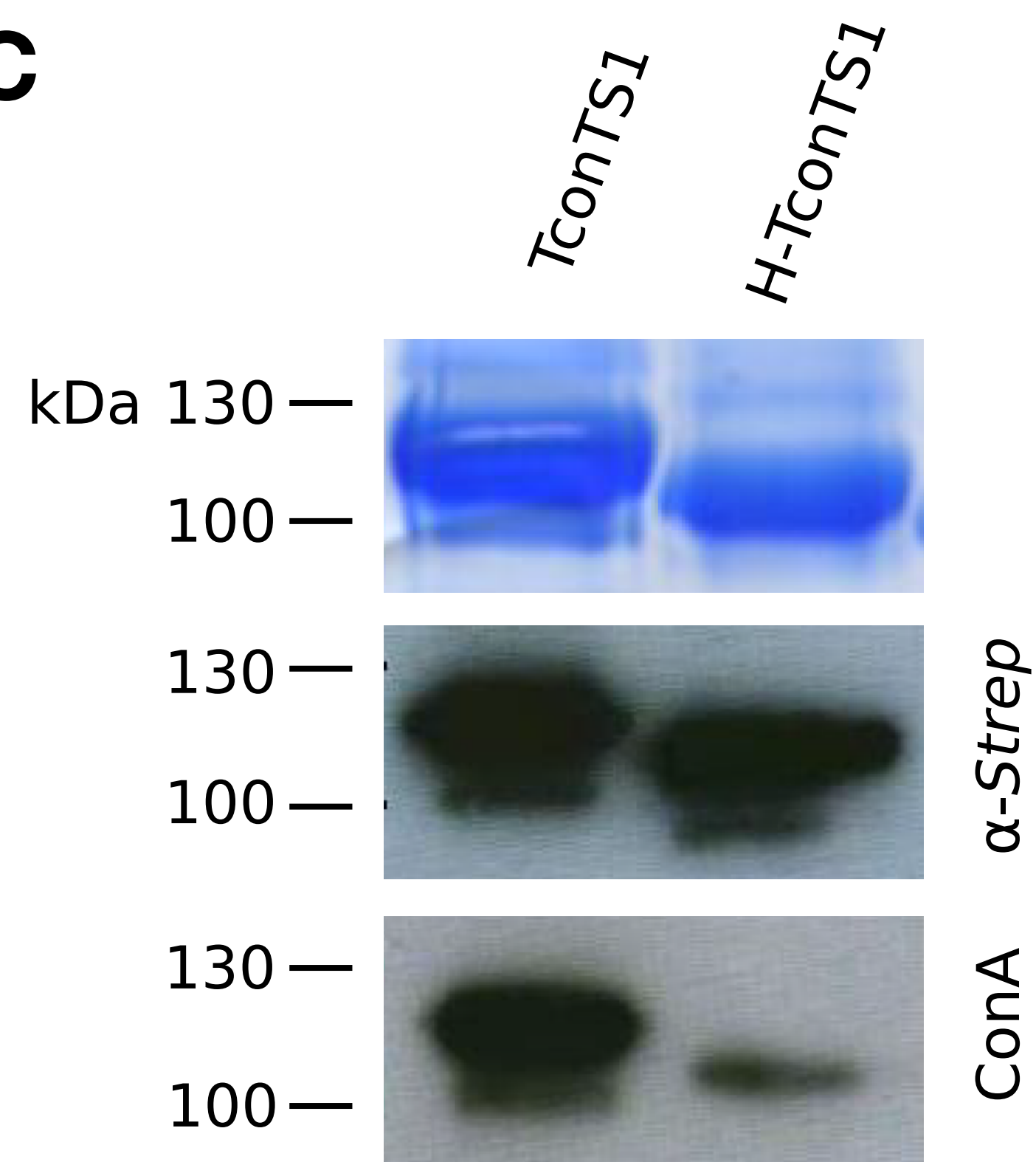**D**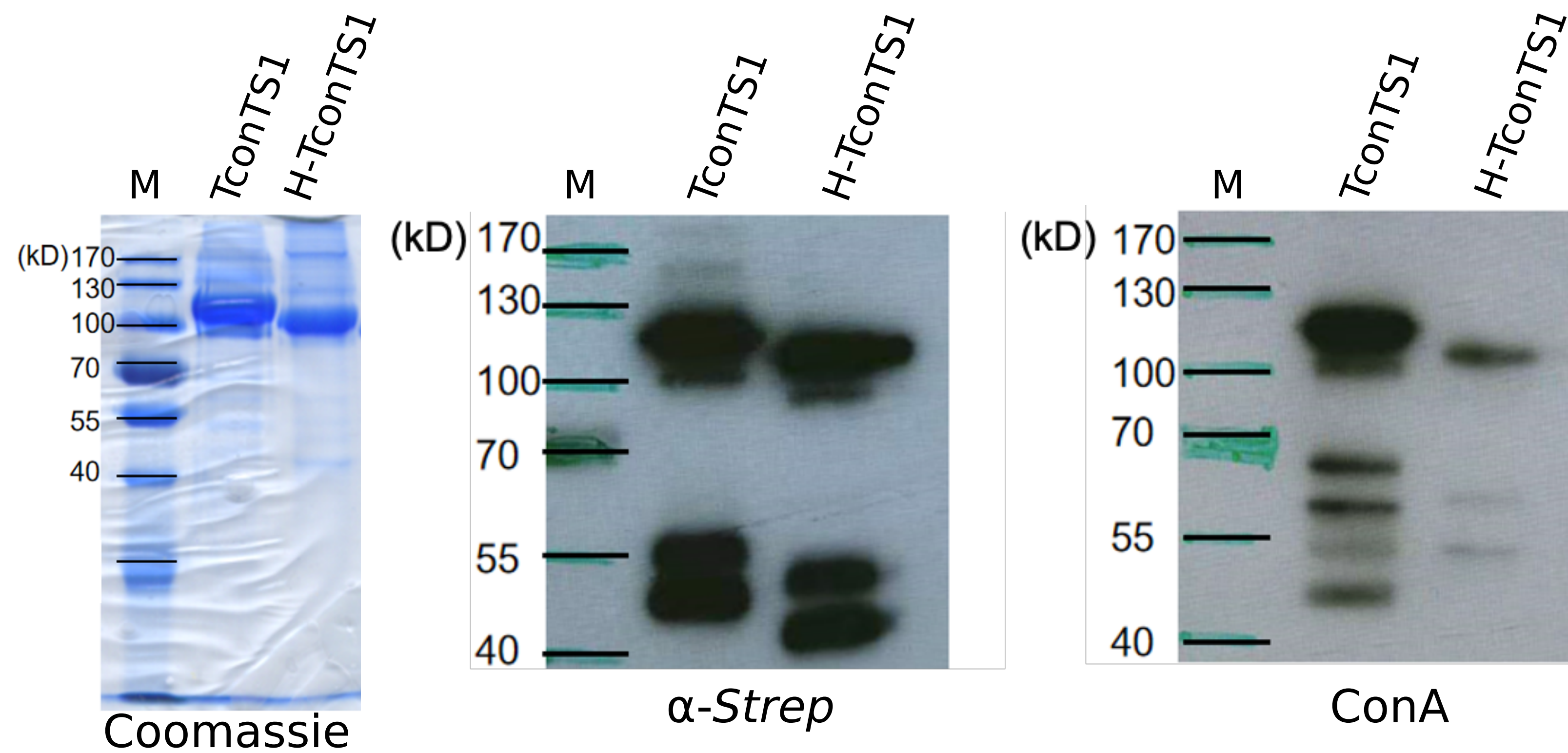

Supplement: Supplemental Figure S4 [file mmc7.pdf]

**A**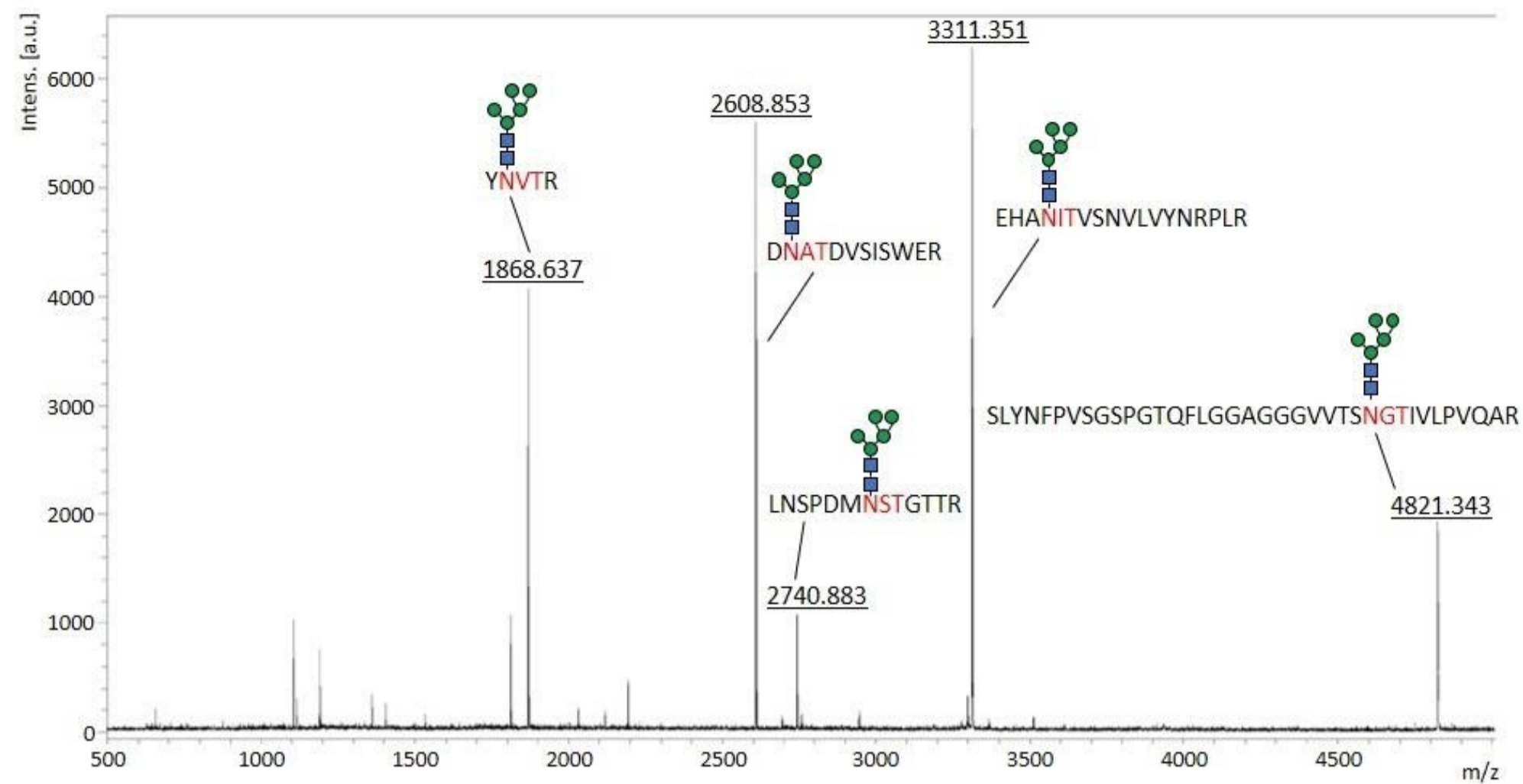**B**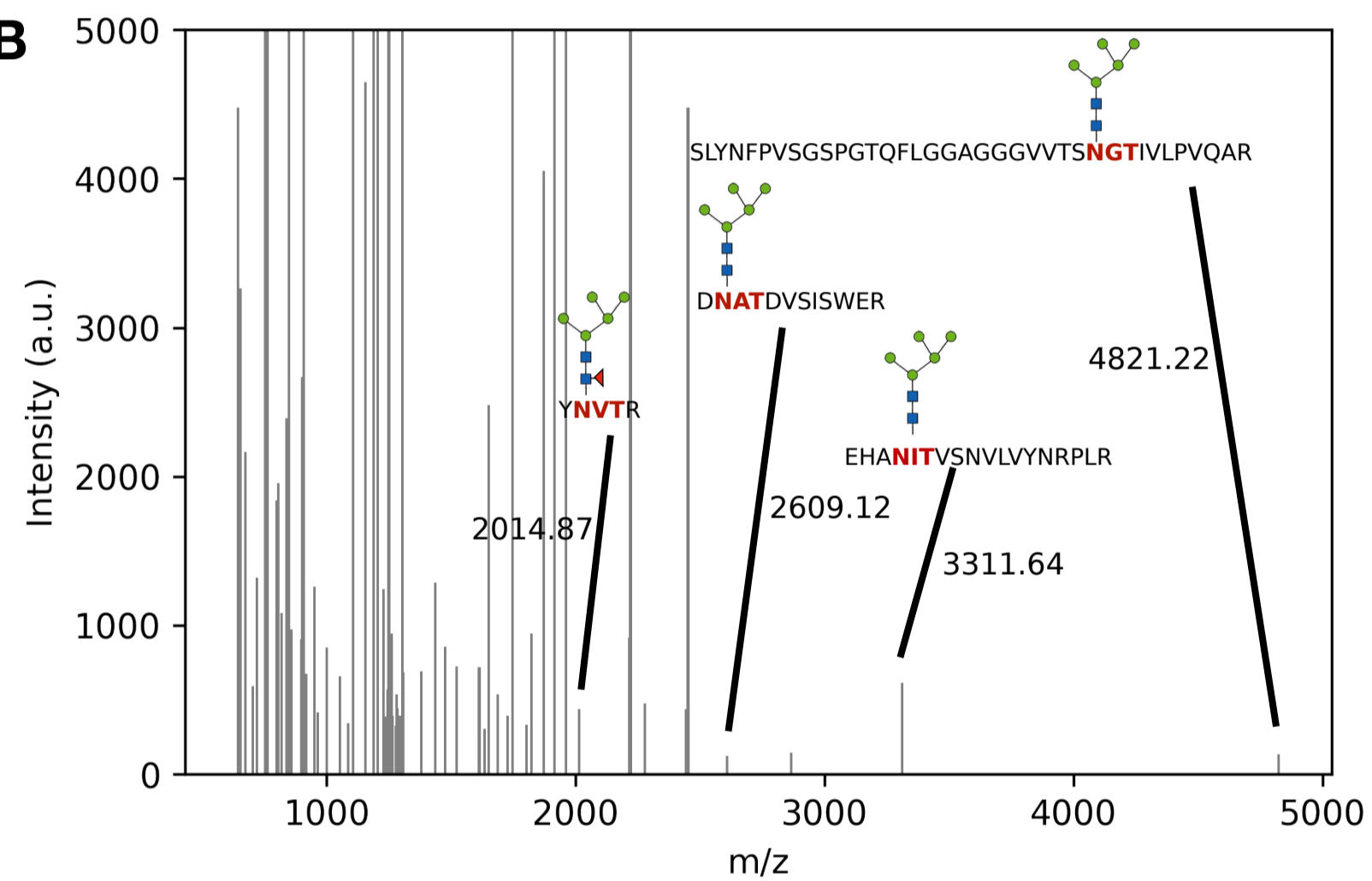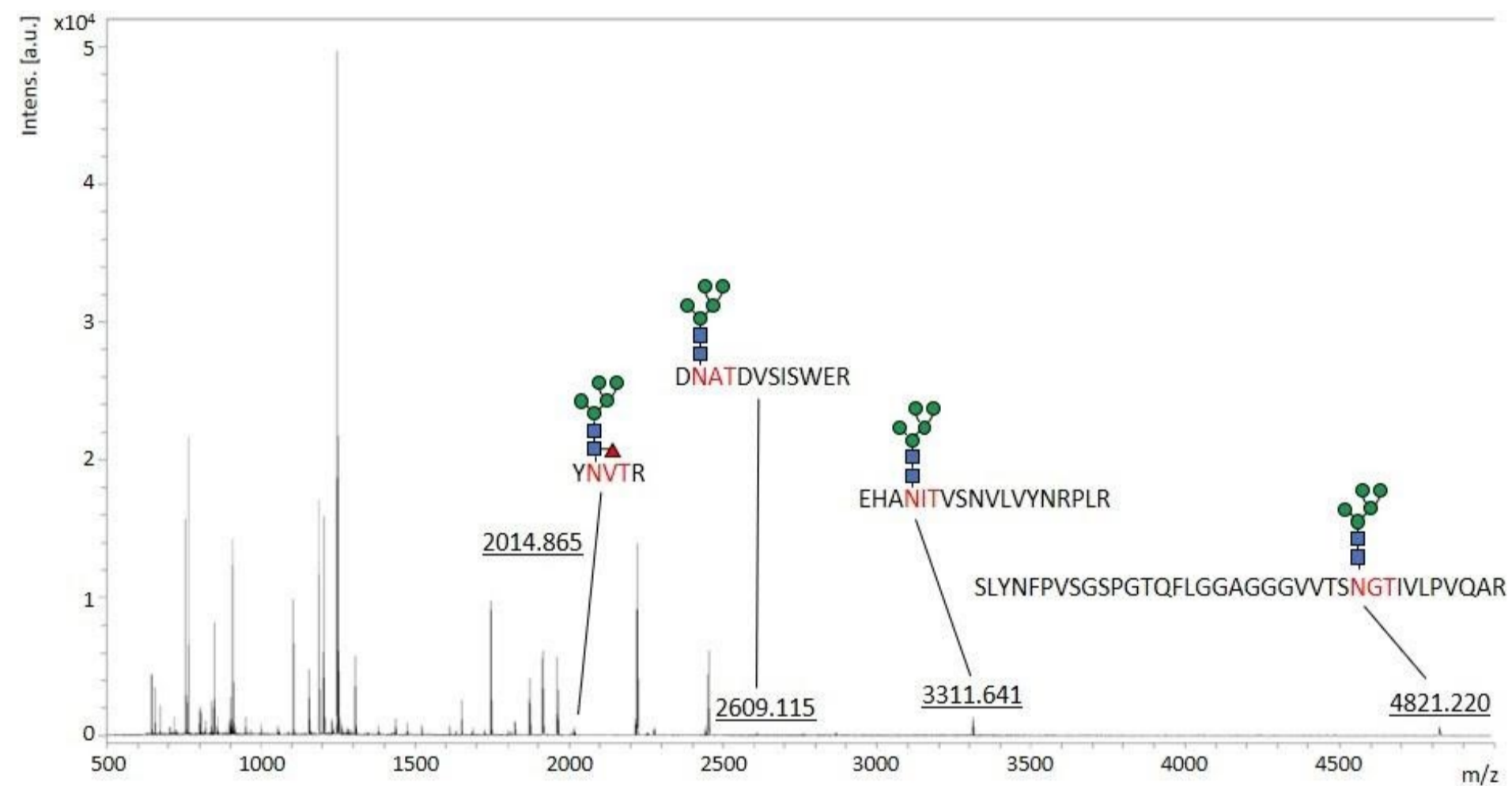**C**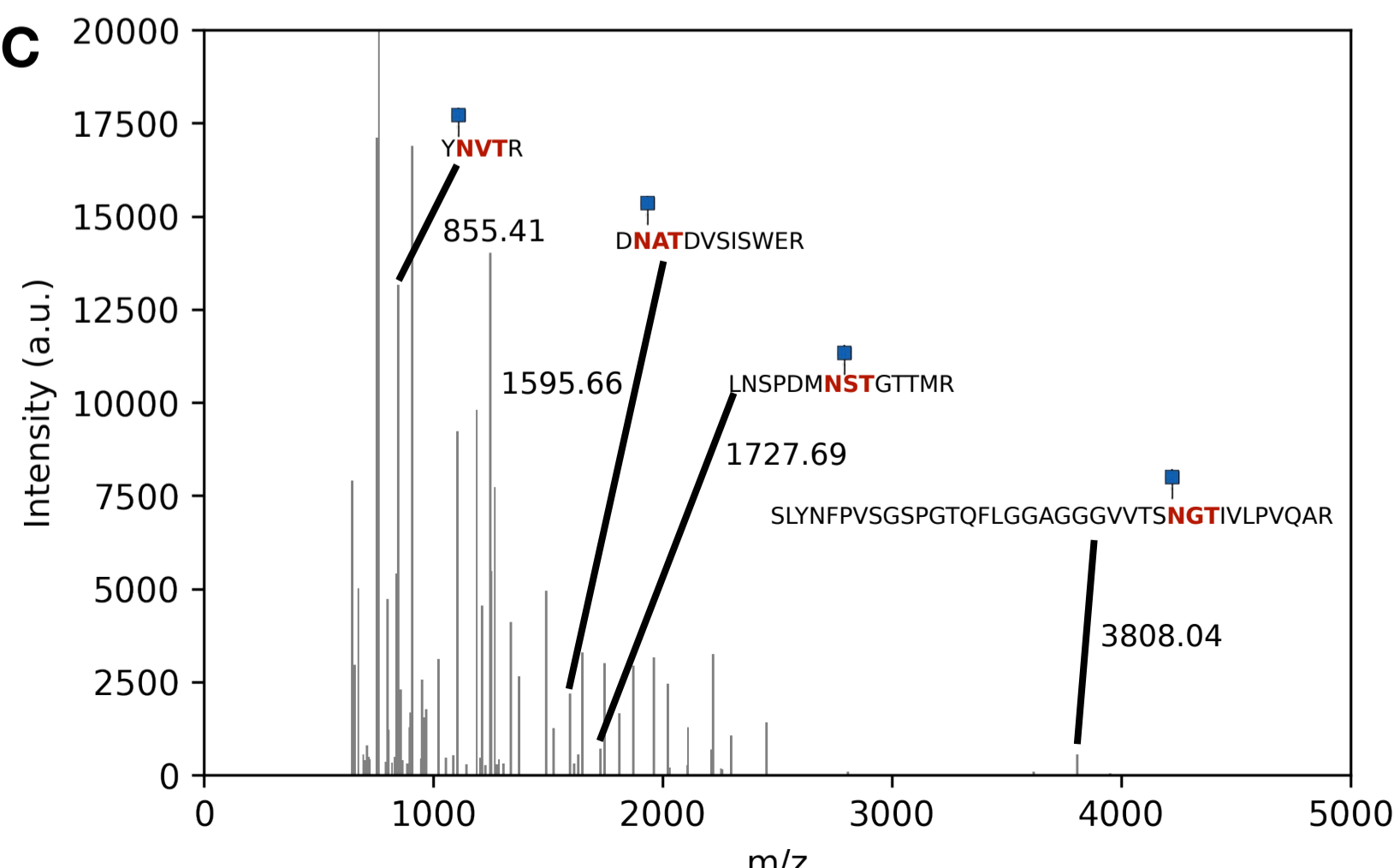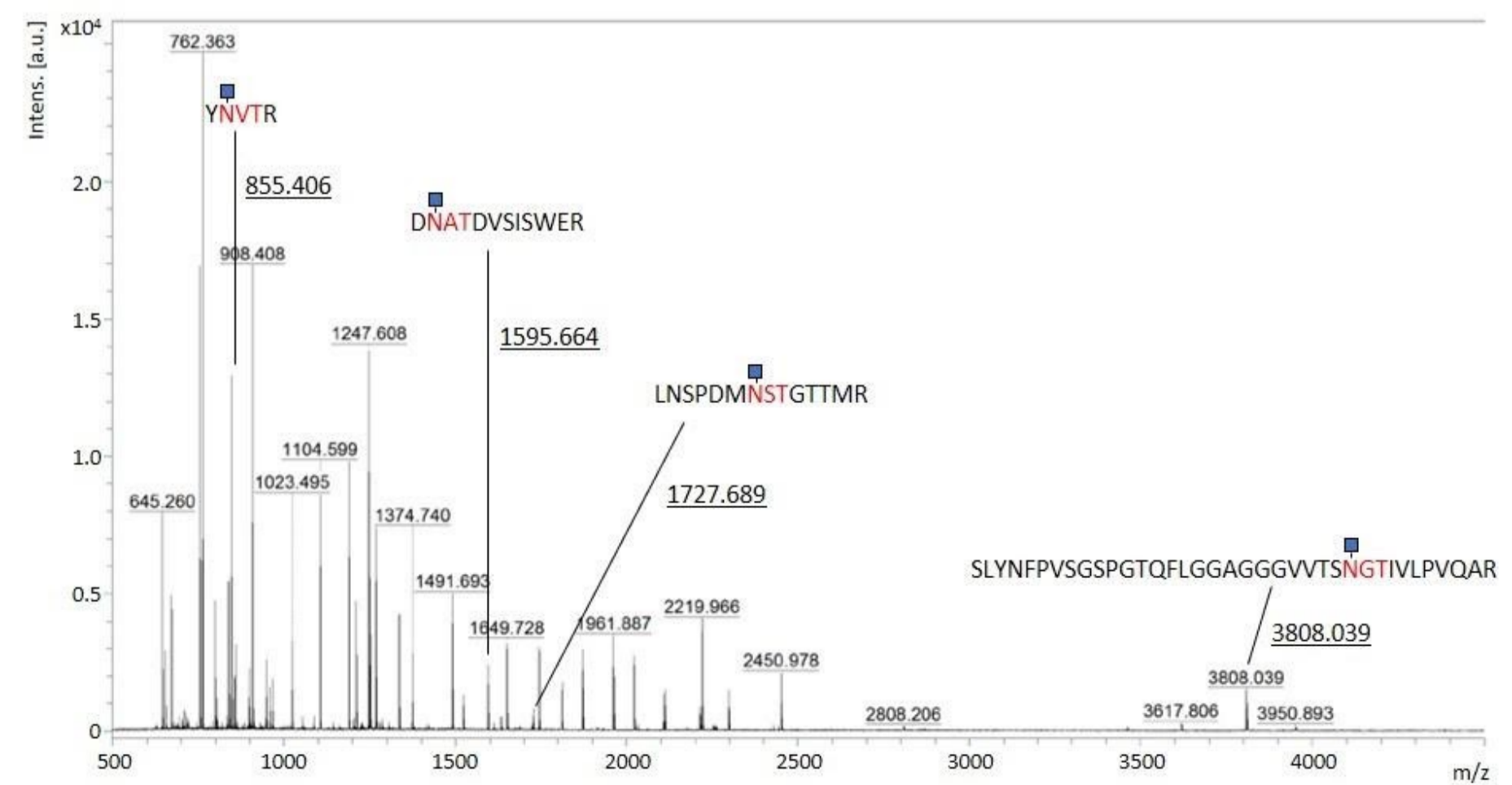

Supplement: Supplemental Figure S5 [file mmc8.pdf]

**A**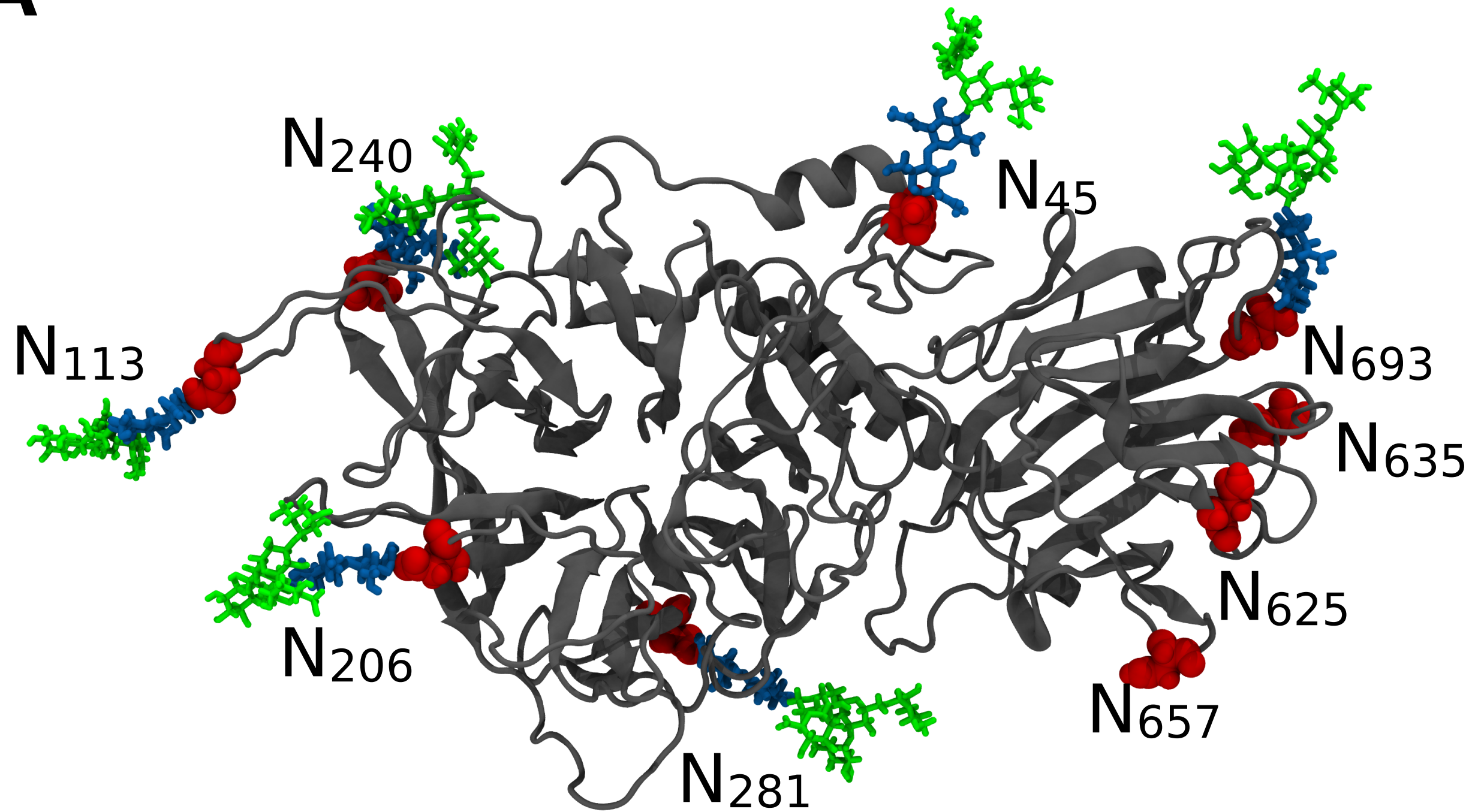**B**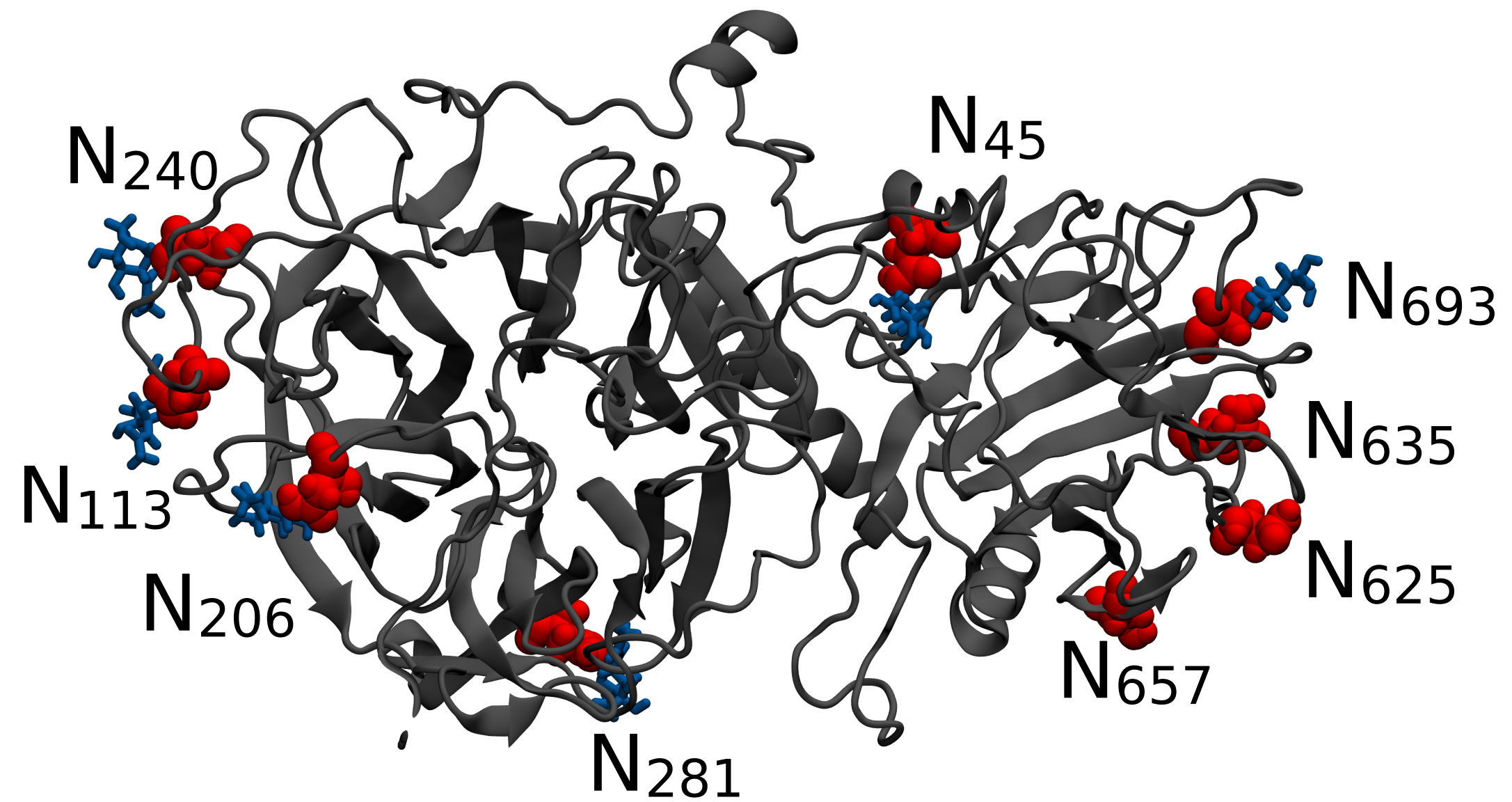

Supplement: Supplemental Figure S7 [file mmc10.pdf]

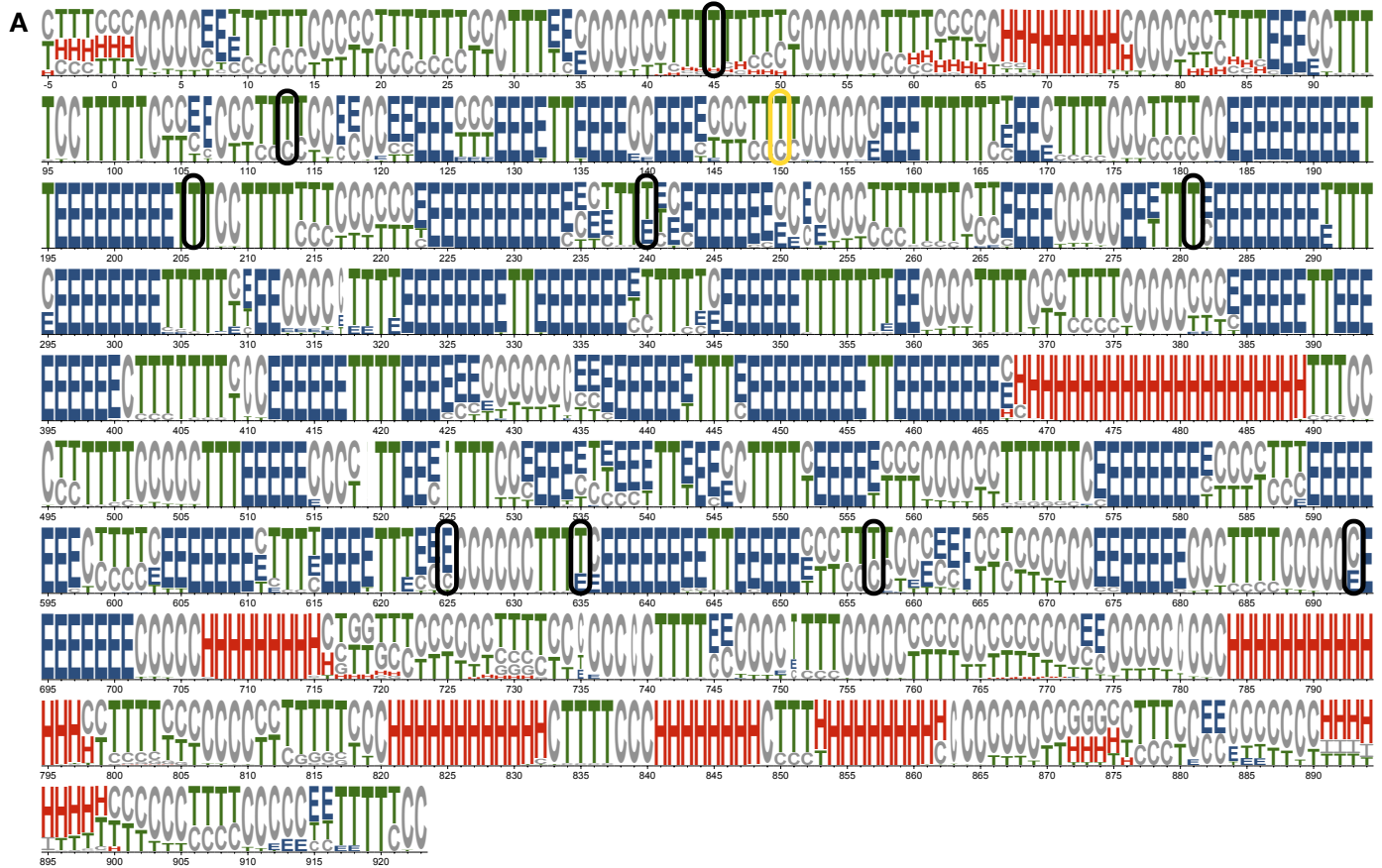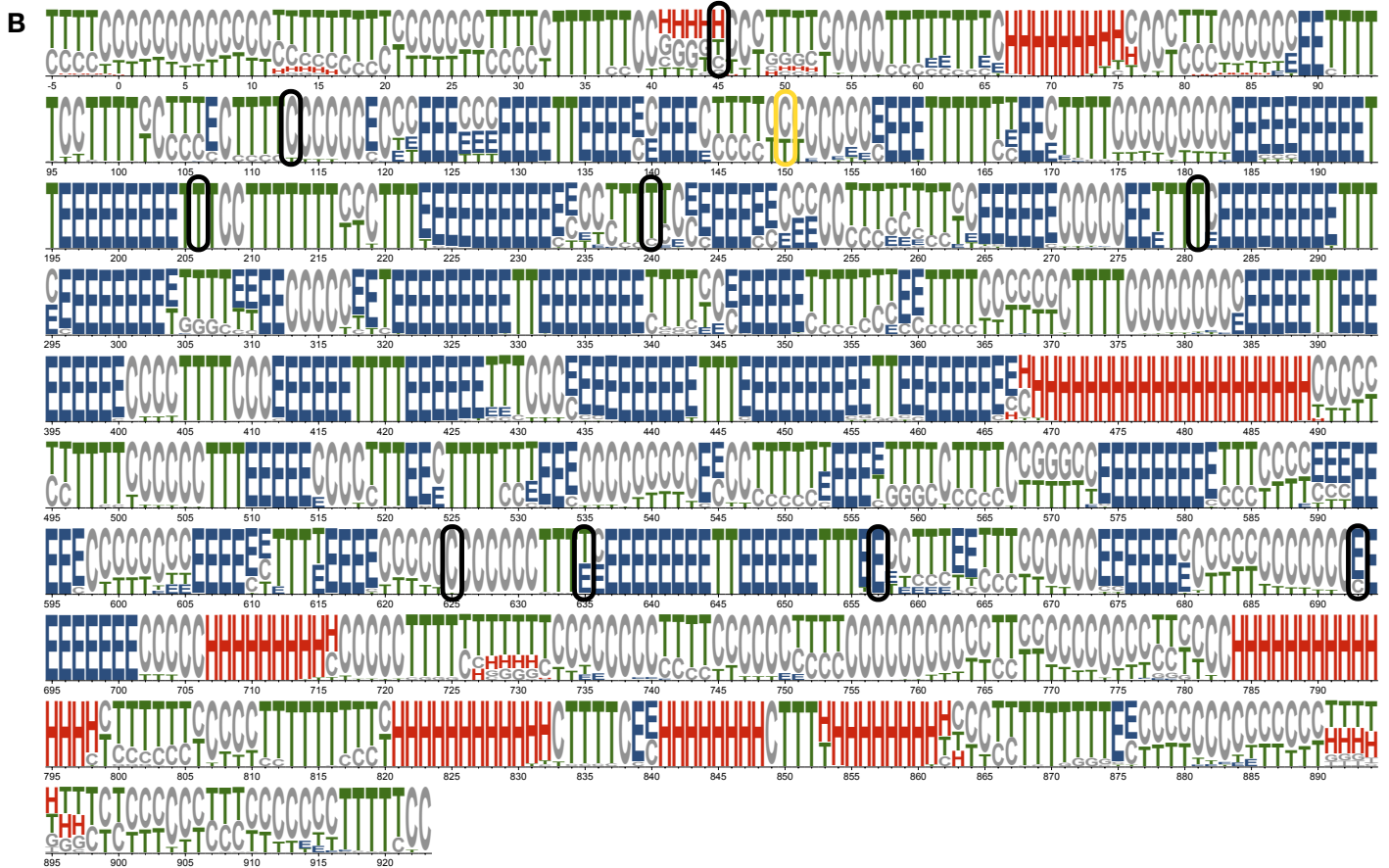

Supplement: Supplemental Figure S8 [file mmc11.pdf]
