# Supplementary material for: N-glycosylation modulates enzymatic activity of Trypanosoma congolense trans-sialidase
Source: J Biol Chem. 2022 Aug 20;298(10):102403. doi: 10.1016/j.jbc.2022.102403 (PMC9493392; doi:10.1016/j.jbc.2022.102403)
Supplement: Supporting information [file mmc12.docx]

Supporting information

S1. Amino acid sequences

A recombinant TconTS1 construct was used for protein expression as described by Koliwer-Brandl et al. (34) (Fig. S1A). In the construct, a transin signal sequence replace the native N-terminal signal peptide, and a SNAP-*Strep* tag at the C-terminus the GPI anchor sequence (Fig. S1B). This recombinant protein allows for the purification from cell media due to its secretion from mammalian cells and a facilitated detection by the *Strep*-tag. Details about construct building and expression are given in reference (34). An overview of the recombinant construct is given in Figure S2.

An alignment of several TS sequences from different species (Fig. S3) shows the overall distribution of *N*-glycosylation sites marked by violet triangles. Overlaps of *N-*glycosylation positions are especially visible for the sites N113 and N206, according to the TconTS1b nomenclature labelling. These sites are indicated by red squares and are identical in up to 3-4 different TS species. Namely, N113 is conserved between TranSA, TvivTS1 and TconTS1b and N206 between TcruTS, TranSA, TbruTS and TconTS1b. This consistency of *N*-glycosylation sites might hint at conserved features, which could be important for enzyme structure or function. Overall, all TS species investigated here harbor at least two *N*-glycosylation sites.

S2. Coomassie gels and blots

In order to verify and assess the efficiency to deglycosylate TconTS1 using EndoH_f_, several techniques have been used including SDS-PAGE with Coomassie staining, western blotting using an anti-*Strep*-tag antibody and lectin blotting using ConA for detection of high-mannose type *N*-glycans. TconTS1 samples have always been incubated at the same temperature and for the same time as H-TconTS1, only lacking EndoH_f_.

First, EndoH_f_ treatment has been performed for 4 hours (Fig. S4A), leading to a clear shift of H-TconTS1 from ~120 to ~110 kDa in Coomassie and western blot analysis, indicating a mass loss due to removal of high-mannose type *N*-glycans. The ConA blot, however, indicated that complete removal of *N*-glycans was not achieved. A band was still visible at 110 kDa for H-TconTS1, although much weaker compared to TconTS1. The original and fully displayed gels of Fig. S4B also showed the presence of EndoH_f_ at ~70 kDa in H-TconTS1, but not in TconTS1 samples.

Due to the lack of complete *N*-glycan removal after 4 hours of incubation, different incubation times of up to 48 hours or higher amounts of EndoH_f_ enzyme were tested. Complete removal of high-mannose type *N*-glycans could still not be achieved, probably due to low accessibility of the *N*-glycan for EndoH_f_ (data not shown).

In the end, we decided for an overnight incubation of 16 hours with EndoH_f_ and used these samples in all following experiments like MALDI-TOF MS, enzyme assays and circular dichroism (Fig. S4C). Also, in these blots a clear reduction of mass for H‑TconTS1 bands is visible. Bands are not as clear and thin as in Figure S3A, as around 5 times more sample was loaded on all gels to detect possible degradation products. An overloading/overexposure was therefore visible. The thickness of bands might additionally result from a certain *N-*glycan heterogeneity in the samples, resulting in different migration behaviors. Original gel and blots in Fig. S4D of Fig. S4C indicate several bands in western and ConA blots at masses around 40 – 70 kDa. These bands are likely fragmentation products of TconTS1, as they could still be detected with the anti-*Strep*-Tag antibody, indicating the presence of the *Strep*-tag, and harbor *N*-glycans as detected in the ConA blot. However, the Coomassie gel in Fig. S3D clearly showed the TconTS1 band at 120 kDa and H-TconTS1 band at 110 kDa as the most prominent proteins, indicating a mostly intact protein in the sample. These results also underline the sensitivity of western and ConA blots for small amounts of protein, as the western blot showed the same signal intensity for bands around 120 kDa and around 50 kDa, although the Coomassie gel unambiguously demonstrates the different protein amount for the different bands.

S3. MALDI – TOF MS

TconTS1 harbors nine potential *N*-glycosylation sites, five in the *N*-terminal catalytic and four in the lectin-like domain. MALDI-TOF MS was employed to assess the glycosylation status of each *N*-glycosylation site. The setup of the Bruker Autoflex Speed device used in this study does not offer in-source fragmentation.

Detailed instrument settings for this analysis are given in Table S1. Furthermore, the software Flex analysis 3.4 (Bruker Corporation, USA) was employed for data evaluation. Monoisotopic peaks were identified with Sophisticated Numerical Annotation Procedure (SNAP) algorithm and a signal to noise ratio of 6. Further settings included baseline subtraction (TopHat) and spectra smoothing (algorithm: Savitzky Golay). The online tools PeptideMass (89), FindPept (90) and GlycoMod (91) (used from 2019-2021) were used to determine (glyco)peptides as [M+H]^+^ ions and glycan structures assuming especially the presence of high-mannose type *N*-glycans as identified on proteins expressed in CHO Lec1 cells (45). Differences in m/z of 0.1 Da were accepted for identification. An exception was the analysis of TconTS1 (trypsin-digested, ConA purified) since m/z ratios of all identified glycopeptides with *N*-glycosylation sites differed by ca. 0.2 Da most likely due to the calibration. Thus, 0.2 Da was accepted for this analysis which was also the default setting of GlycoMod (91). Peaks identified in negative controls N1 and N2 were excluded from sample peak lists. In addition to glycan residues, modifications like oxidation of methionine and alkylation of cysteine were considered during peak assignment.

In order to analyze (H-)TconTS1 with MALDI-TOF MS, it was necessary to cut the enzyme into shorter peptide and glycopeptide fragments via protease digestion. In case an asparagine residue in the N-X-S/T motif of a certain glycopeptide is glycosylated the m/z ratio increases by exactly the mass of the conjugated *N*-glycan relative to the non-glycosylated peptide.

Three different approaches were used for peak assignment, as instrument settings did not allow for peak fragmentation and hence an unambiguous assignment of peaks. However, we believe that the combination of following experiments is suitable for cross-validation of identified peaks:

1. The usage of trypsin or chymotrypsin for generation of glycopeptides yields altered peptide profiles due to different specific protease recognition sites (Fig. S5B). The same *N*-glycosylation site can therefore be identified from different glycopeptides (different size/mass). If glycopeptides can be identified from trypsin and chymotrypsin digested samples, there is a high possibility that the assigned peak is the predicted glycopeptide. Along this line, if the mass increase is identical for glycopeptides containing the same *N*-glycosylation site when compared to the non-glycosylated peptide, this is a strong indication that this site is occupied with a defined *N*-glycan.

2. Subsequent ConA-sepharose purification of glycopeptides yielded only glycopeptides with high-mannose type *N*-glycans in the sample. As a consequence, only glycosylated fragments can be measured and identified. A further advantage of this approach is a reduced peak complexity in spectra as most peptide fragments are filtered out (Fig. S5A).

3. Further, H-TconTS1 was digested with trypsin and chymotrypsin. This step is an additional validation for the presence of *N*-glycans as EndoH_f_ cleaves the *N*-glycan tree in the chitobiose core, leaving one GlcNAc residue to the *N*-glycosylation site. These peptides fragments in H-TconTS1 samples can be detected as they exhibit a mass difference of m/z 203.08 (one HexNAc) relative to the non-glycosylated peptide. A remaining GlcNAc residue indicates the presence of a high-mannose type *N*-glycan at that site before removal by EndoH_f_ (Fig. S5C).

A detailed list of all assigned peaks with theoretical and measured masses is given in Table S2. For a better overview Table 1 has been generated, separating results by the three different approaches employed for cross-validation of *N*-glycan detection and are discussed in detail in the following paragraphs.

Regarding results summarised in Table1, when using trypsin for protease digestion, all identified glycopeptides (N113, N206, N240, N281, N693) could also be found deglycosylated in H-TconTS1 with a residual HexNAc, indicating a reliable assignment of peaks. These results can be further underlined as the same glycopeptide fragments could be assigned in ConA sepharose purified samples, too. Glycopeptide fragments with high-mannose type *N*-glycans were also identified in H-TconTS1 samples, indicating an incomplete deglycosylation process. However, these fragments could only be identified in ConA sepharose purified samples but not in the complete trypsin digest. This indicates a low frequency of these hypoglycoslyated peptides after treatment with EndoH_f_. Identifying site N240 in a non-glycosylated and glycosylated state indicates glycosylation heterogeneity, which is possible when enzymes of the glycosylation machinery do not have temporal or spatial access to certain sites.

Looking at the chymotrypsin protease digestion, sites N45 and N206 can again be unambiguously defined in glycoproteins, harboring high-mannose type *N*-glycans in TconTS1 and one HexNAc in H-TconTS1. At N113, high-mannose type *N*-glycans were found in TconTS1 but also in H-TconTS1 samples indicating resistance to EndoH_f_ cleavage. The sites N113, N206, and N693 were also identified in a non-glycosylated state in TconTS1 or H-TconTS1 samples. N625 was exclusively found non-glycosylated and never with high-mannose type *N*-glycans or one HexNAc residue, indicating that this site might not harbor *N*-glycans at all. Chymotrypsin digestion in combination with ConA sepharose purification underlines the glycosylation status at N45, N206 and N657 as mainly Man_5_GlcNAc_2_ were identified at these sites. Additionally, site N657 was found with a residual HexNAc in simple chymotrypsin digestion of H-TconTS1, being in agreement with the Man_5_GlcNAc_2_ identified in TconTS1. The presence of high-mannose type *N-*glycan at position N206 in H-TconTS1 samples confirm the results obtained from ConA sepharose purified and trypsin-digested samples towards an incomplete deglycosylation at this site using EndoH_f_.

It seems likely that different proteases produce certain (glyco)peptides with a higher frequency and that miscleavages due to possible protease resistance of glycosylated protein at some sites might also play a role since certain peptides were neither detected in the unmodified nor in a modified status, e. g. with oxidized methionines or alkylated cysteines.

Only a few glycopeptides were identified with only one approach, such as residual high-mannose type *N*-glycans after EndoH_f_ treatment at position N113 and N693 or fucosylated Man_5_GlcNAc_2_ at N206 in TconTS1. We assume that these glycopeptides were rather rare in samples and were therefore not identified with different analyses. As these results are ambiguous, they were not regarded further in this study.

In none of these experiments we could identify a leucine-rich peptide containing the *N*-glycosylation consensus sequence N-E-S (662-664), neither in the glycosylated nor in the non-glycosylated form. The peptide was not detectable also when the mass lists were analyzed for different *N*-glycans and post-translational modifications. Among the many different proteases which are suitable for MALDI-TOF MS analysis, the leucine-rich fragment only contained cleavage sites for chymotrypsin. However, the protease cleaves at the carboxyl side of different amino acids (Y, F, W, L, M), but the cleavage efficiency is rather low after leucine and methionine. It is therefore possible that the leucine-rich fragment was not sufficiently cleaved by chymotrypsin and thus too large to be detected. Another possibility is that the fragment has been digested into many different peptides in quantities below the detection limit.

S4. Circular dichroism experiment

TconTS1 and H-TconTS1 were measured in circular dichroism experiments in a wavelength region from 190 – 250 nm. The secondary structural composition of proteins can be predicted from these spectra by the webserver BeStSeL (66, 67), as final circular dichroism spectra consist of additive signals from secondary structure elements like α-helices or ß-sheets. Table S3 summarizes the extracted secondary structure composition from circular dichroism spectra in Fig. 4A, revealing no significant differences between TconTS1 or H-TconTS1. These results indicate that EndoH_f_ treatment and the removal of *N*-glycans from the protein surface have not influenced the secondary structural composition of H-TconTS1.

S5. Molecular dynamics simulations

Due to missing experimental structures of TS from *T. congolense*, the atomistic structure of TconTS1 was derived using the I-TASSER web server for protein structure and function predictions (69, 70). The engineered SNAP-*Strep* was included for consistency and better comparison with experimental data. The threading algorithm mainly employed TranSA (PDB entry: 2agsA, 2A75) as well as TcruTS (PDB entry: 1ms9) as templates. Validation of the homology modelled TconTS1 was performed by an amino acid sequence alignment of recombinant TconTS1 with TranSA (PDB entry 2ags) and TcruTS (PDB entry: 1ms9) revealing that 10 out of 14 amino acids predicted to be important for enzymatic activities are conserved among all compared models (Fig S6A). Furthermore, 2 of the remaining 4 sites are conserved between TconTS1 and TcruTS (Y211, P379) and only the left over 2 not conserved in TconTS1 (A325, Y408). It needs to be noted that especially Y408, part of the lactose holder pair in the binding site, is a tryptophan in TranSA and TcruTS and therefore both amino acids resemble due to their hydrophobic character. Coloring of the atomistic structure of TconTS1 by the amino acid sequence alignment from Figure S6A is given the impression of most conserved residues to be located in ß-sheet or α-helix regions (Fig S6B). Amino acids of loop regions seem to be less conserved, probably also being less important for the overall structural folding and function of the enzyme. Structural alignment of TconTS1 with TranSA and TcruTS reveal a high similarity of all models with respect to the secondary and tertiary structure (Fig S6C). Only the N-terminal part of TconTS1 is in general longer compared to TranSA and TcruTS and therefore cannot be aligned. Independent prediction of the secondary structure by I-Tasser as well as the inherent thermal mobility of each residue of TconTS1 are akin to that of TranSA and TcruTS (Fig S6D). This is because ß-sheets are dominant in the catalytic and lectin domain, whereas an α-helix is connecting both domains.

Despite an amino acids sequence identity of only 37/38 % between recombinant TconTS1 (with SNAP-*Strep* Tag) and TranSA/TcruTS, the I-Tasser homology model validation suggests a similar secondary structure of TconTS1 compared to other TS from different species and therefore predicts a likewise tertiary model. Conservation of almost all catalytically involved amino acids in TconTS1 further support the idea of structural similarity to the other TS. The I-Tasser confidence score of the recombinant TconTS1 model was given with -2.99 (range -5 to 2), where a higher value signifies a higher confidence. It is, however, necessary to note that the artificial SNAP-*Strep* domain is included in this assessment, and an analysis of only the native TconTS1 results in a confidence score of -0.63, stating a much higher reliability. A confidence score of above -1.5 means that more than 90% of the predictions are correct and therefore our TconTS1 models is considered to be predicted with an overall correct fold (69). Final objections of model accuracy can however only be addressed with structure determination techniques like X-ray crystallography in the future.

Homology modelled TconTS1 was further modified by CHARMM-GUI Glycan Modeler (www.charmm-gui.org) to construct a glycosylated variant termed TconTS1 representing the highest degree of *N*-glycosylation that has been detected by MALDI-TOF MS experiments namely for sites N45, N113, N206, N240, N281 and N693 (Fig S7A). Man_5_GlcNAc_2_ was chosen as a model glycan tree for all sites, as it is the most abundant found form of *N*-glycans in CHO Lec1 cells (45). The EndoH_f_ treated variant was generated with single GlcNAc residues at sites N45, N113, N206, N240, N281 and N693 (Fig S7B), which were previously found to be glycosylated. No remaining high-mannose type *N*-glycan trees were included in the modelled enzyme, although incomplete deglycosylation has been observed in experiments for certain sites. For consistency, the enzyme is still termed H-TconTS1. It was the aim to represent the highest degree of deglycosylation, by attaching residual GlcNAc at the asparagine residues in N-X-S/T motives.

Qualitative comparison of secondary structural differences between TconTS1 and EndoH_f_ treated H-TconTS1 were not only addressed experimentally by circular dichroism experiments, but also further verified by MD simulations. Therefore, the assignment of secondary structure elements was tracked over time for each residue (Fig. S8). In total, it can be said that the overall secondary structure of TconTS1 and H-TconTS1 is comparable over the simulated time, as α-helices in the N-terminal region (position 67 – 76), between the catalytic and lectin domain (position 468 – 489) as well as in the C-terminal SNAP-*Strep* region (position from 707 onwards, Fig. S1A) are consistent. The same applies to the shorter but more frequently observed ß-sheet regions. These simulated results support the findings of circular dichroism experiments, in which no changes in secondary structure elements could be observed, too.

Additionally, it can be seen that *N*-glycosylation sites are mostly situated in coil and turn secondary structural motifs, positioned next to ß-sheet regions. The same applies to residue D150, positioned in-between two ß-sheet regions, with at least 5 amino acids assigned to coil and turn elements directly next to each other. These observations hint at a certain flexibility of *N*-glycosylation sites and amino acid D150, not involved in extensive secondary structure motives of α-helices or ß-sheets.

Tables

**Table S1:** Detailed settings of the MALDI-TOF autoflex^TM^ speed method used for identification of glycosylated and non-glycosylated peptides from TconTS1 and H-TconTS1.

**Table S2:** List of peptides with *N*-glycosylation sequences identified in MALDI-TOF mass spectra. TconTS1 and H-TconTS1 were digested with trypsin and chymotrypsin and glycopeptides were optionally purified with ConA adsorbent. Mass spectra were analyzed for the presence of peptides with the N-X-S/T recognition sequence and for the corresponding glycopeptides with high-mannose type *N*-glycans or HexNAc residues (H-TconTS1 only). Ions measured were only charged once ([M+H]^+^). Masses of glyco(peptide) fragments including modifications like oxidation of methionine and alkylation of cysteine could not be detected. *(Glyco)peptides only identified once during multiple analyses.

**Table S3:** Secondary structure elements of TconTS1 and H-TconTS1, calculated using BeStSeL from the respective circular dichroism spectra (Fig. 4A).

**Figure S1**: A) Amino acid sequence of recombinant TconTS1 (see Figure S2) used in this study. The signal peptide sequence of the native protein (B, GenBank ID HE583284) was replaced by a transin sequence for efficient cell secretion and the GPI anchor sequence was replaced by a SNAP- and *Stre*p-tag for protein labelling and purification. To be congruent with previous literature about TconTS1, the amino acid numbering of the native protein was used in the text. A schematic structure of the recombinant truncated protein is given in Figure S2. *N*-glycosylation sites are underlined in red.

**Figure S2:** Naturally occurring TS are composed of a catalytic and lectin-like domain (CD, LD) linked by an α-helix. The recombinant protein construct of this study contained the TconTS1 coding sequence without the naturally occurring signal peptide and GPI anchor sequence and was additionally supplied with an N-terminal transin sequence for cell secretion and a C-terminal SNAP- and *Strep*-tag for protein labelling and purification, respectively.

**Figure S3:** Sequence alignment of TS from *T. cruzi* (TcruTS, GenBank ID AAA66352), *T. brucei brucei* (TbruTS, GenBank ID AAG32055), *T. congolense* (TconTS1b, GenBank ID HE583284), *T. vivax* (TvivTS, GenBank ID CCD21087) and the closely related sialidase from *T. rangeli* (TranSA, GenBank ID AAC95493) was generated with the ClustalW Alignment tool of the software Geneious Pro 5.5.9 employing the BLOSUM matrix with a gap opening cost of 10 and gap penalty cost of 0.1. Conserved glycosylation sites are indicated (red box).

**Figure S4:** A) Coomassie staining (upper panel with 600 ng), western blot analysis using an anti-*Strep*-tag antibody (middle panel with 400 ng of protein in each slot) and ConA (lower panel with 100 ng of protein) of TconTS1 and H-TconTS1 after 4 h of EndoH_f_ treatment. Exposure time of western blot is 5 sec and 60 sec for the ConA blot. Identical to Fig. 2A. B) Full blots used in A) with original marker bands (M). Blots were performed before purification of H-TconTS1 by *Strep*-Tactin Sepharose affinity chromatography to remove the EndoH_f_ enzyme. A positive control of EndoH_f_ is loaded for the Coomassie gel and western blot. Faint bands in the Coomassie gel at 70 kDa correspond to the EndoH_f_ enzyme. The fainter band below 70 kDa­ in all three lanes of the commassie stained gel most likely results from contamination in the sample buffer. C) TconTS1 and H-TconTS1 (EndoH_f_ treated for 16 h) were analyzed by SDS-PAGE with subsequent Coomassie staining (upper panel with 5 µg), by western blot analysis using an anti-*Strep*-tag antibody (middle panel with 1 µg) and by lectin blotting using ConA (lower panel with 1 µg). western and ConA blots were exposed for 5 and 10 sec, respectively. D) Original blots for C) with original marker bands (M).

**Figure S5:** A) Original and fully unzoomed MALDI-TOF mass spectrum of ConA-purified tryptic peptides of TconTS1 from Fig. 2B. B) MALDI-TOF MS analysis of protease-digested TconTS1 identified glycosylated and non-glycosylated peptides. C) MALDI-TOF MS analysis of H-TconTS1 confirmed high-mannose type *N*-glycosylation of several glycopeptides as the EndoH_f_ treatment only cleaves these specific glycans resulting in residual HexNAc. Peak lists from spectra were extracted and plotted with python and annotated with corresponding masses and glycopeptide fragments, respectively. They can be compared to their original, unzoomed spectra on the right (B/C). Monosaccharide symbols follow the Symbol Nomenclature for Glycans (SNFG) (92).

**Figure S6:** A) Amino acid sequence alignment of recombinant TconTS1, TranSA (PDB entry: 2ags) and TcruTS (PDB entry: 1ms9) by ClustalW using the bioinformatics analysis tool MultiSeq (93) implemented in VMD. Fully conserved amino acids are depicted in blue, partially conserved in white and not conserved in red. Residues of the catalytic domain, which are considered to be important for enzymatic activity are surrounded by a black box (39, 51). Residue numbering is in correspondence with the native TconTS1 sequence. B) 3D structure of TconTS1 in a cartoon style, where coloring of each amino acid is in correspondence with A) The C-terminal SNAP-Strep-Tag is not shown for simplicity. C) Structural alignment of TconTS1, TranSA (PDB entry: 2ags) and TcruTS (PDB entry: 1ms9) by VMD represented in cartoon style. Coloring is in accordance with the Q factor of each residue, where Q is a metric for structural homology implemented in VMD. Blue is referring to 100 % structural identity and a color shift over white to red, a less well alignment. D) Plot of the normalized B-factor, representing the inherent thermal mobility of each residue with indication of predicted secondary structural elements, generated by I-Tasser (69, 70).

**Figure S7:** Atomistic structure model of TconTS1 A) and H-TconTS1 B) including Man_5_GlcNAc_2_ or GlcNAc residues at selected *N*-glycosylation sites respectively. Representation and coloring as in Figure 5.

**Figure S8:** A) Secondary structure of TconTS1 averaged over 500 ns of classical MD. B) Secondary structure of H-TconTS1 averaged over 500 ns of classical MD. Size of letters is corresponding to the probability of secondary structure element occurrence. H = α-helix (red), E = ß-sheet (blue), T = turn (green), C = coil, B = Isolated bridge, I = Pi-helix (grey). Positions of *N*-glycosylation sites are marked with black circles. Position of D150 is marked in yellow. Secondary structure was calculated using the STRIDE module of VMD (94). The figure has been created using the weblogo 3 website (95).
