# Supplementary material for: N-glycosylation modulates enzymatic activity of Trypanosoma congolense trans-sialidase
Source: J Biol Chem. 2022 Aug 20;298(10):102403. doi: 10.1016/j.jbc.2022.102403 (PMC9493392; doi:10.1016/j.jbc.2022.102403)
Supplement: Supplemental Figure S6 [file mmc9.pdf]

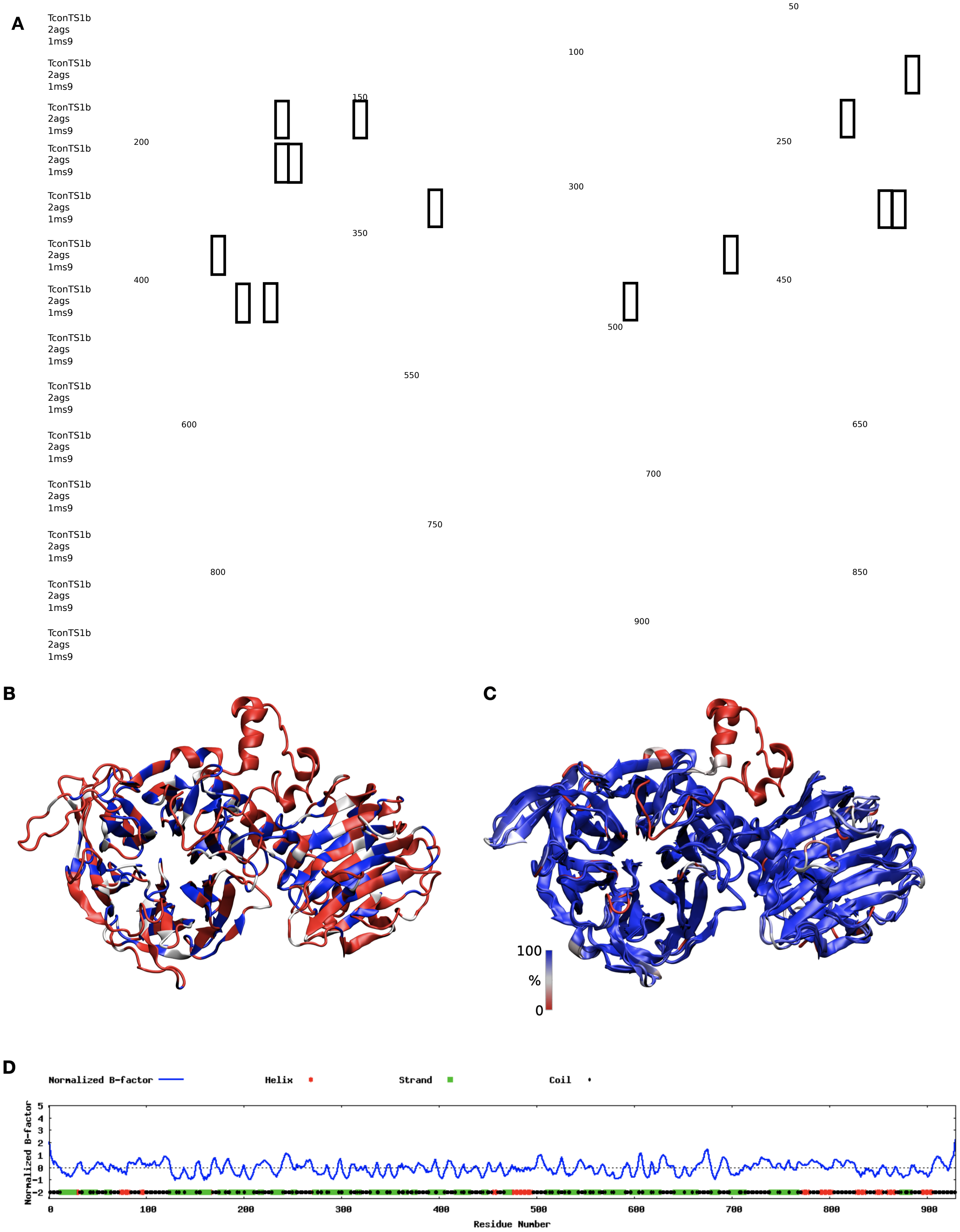
A
TcconTS1b
2ags
1ms9
50
100
150
200
250
300
350
400
450
500
550
600
650
700
750
800
850
900
B
C
100
%
0
D
Normalized B-factor
Helix
Strand
Coil
Normalized B-factor
Residue Number
